# A New Insight Into the Underlying Adaptive Strategies of Euryhaline Marine Fish to Low Salinity Environment: Through Cholesterol Nutrition to Regulate Physiological Responses

**DOI:** 10.3389/fnut.2022.855369

**Published:** 2022-04-14

**Authors:** Yangguang Bao, Yuedong Shen, Xuejiao Li, Zhaoxun Wu, Lefei Jiao, Jing Li, Qicun Zhou, Min Jin

**Affiliations:** ^1^Laboratory of Fish and Shellfish Nutrition, School of Marine Sciences, Ningbo University, Ningbo, China; ^2^Key Laboratory of Aquaculture Biotechnology Ministry of Education, Ningbo University, Ningbo, China

**Keywords:** cholesterol metabolism, endoplasmic reticulum stress, LC-PUFA biosynthesis, low salinity, osmoregulation, oxidative stress

## Abstract

Salinity is an important environmental factor that can affect the metabolism of aquatic organisms, while cholesterol can influence cellular membrane fluidity which are vital in adaption to salinity changes. Hence, a 4-week feeding trial was conducted to evaluate the effects of water salinity (normal 23 psu and low 5 psu) and three dietary cholesterol levels (CH0.16, 0.16%, CH1.0, 1.0% and CH1.6, 1.6%) on osmoregulation, cholesterol metabolism, fatty acid composition, long-chain polyunsaturated fatty acid (LC-PUFA) biosynthesis, oxidative stress (OS), and endoplasmic reticulum stress (ERS) of the euryhaline fish black seabream (*Acanthopagrus schlegelii*). The results indicated that in low salinity, fish fed with the CH1.0 diet improved ion reabsorption and osmoregulation by increased Na^+^ concentration in serum as well as expression levels of osmoregulation-related gene expression levels in gills. Both dietary cholesterol level and water salinity significantly affected most cholesterol metabolic parameters in the serum and tissues, and the results showed that low salinity promoted cholesterol synthesis but inhibited cholesterol catabolism. Besides, in low salinity, hepatic expression levels of LC-PUFA biosynthesis genes were upregulated by fed dietary cholesterol supplementation with contents of LC-PUFAs, including EPA and DHA being increased. Malondialdehyde (MDA) was significantly increased in low-salinity environment, whereas MDA content was decreased in fish fed with dietary CH1.0 by activating related antioxidant enzyme activity and gene expression levels. A similar pattern was recorded for ERS, which stimulated the expression of nuclear factor kappa B (*nf-*κ*b*), triggering inflammation. Nevertheless, fish reared in low salinity and fed with dietary CH1.0 had markedly alleviated ERS and downregulated gene expression levels of pro-inflammatory cytokines. Overall, these findings demonstrate that cholesterol, as an important nutrient, plays vital roles in the process of adaptation to low salinity of *A. schlegelii*, and provides a new insight into underlying adaptive strategies of euryhaline marine fish reared in low salinity.

## Introduction

Salinity is a major environmental stress factor for marine organisms. Change in salinity not only affects body metabolism pathways and osmotic regulation (1), but also affects the physiological and biochemical conditions of marine organisms in various aspects, including growth performance, reproduction development, energy metabolism, and immune system (2–6). However, in recent years, seawater salinity has been continuously decreased because of challenges of tidal fluctuations, extreme weather, melting glaciers, and other factors, which disrupt the balance of the water cycle and lead to the lag of water layer exchange, especially in estuaries (7). Furthermore, the aquaculture production of euryhaline organisms has gradually expanded from coastal environments to inland areas to take advantage of cleaner water resources and economic development in these areas (8). In response to low salinity stress, marine fish must have developed specific adaptations and biological characteristics (9). Many studies have shown that gills are the main functional tissues in osmoregulation of marine fish, which can regulate the balance of osmotic pressure through the action of biological enzymes, regulation of hormones, utilization of ion transport channels, and systematic conduction of biological signals (10). Currently, studies are mainly focused on the effects of water salinity on tissues and organs, growth and development, physiological metabolism, and other aspects of fish (11). Although there are many studies have reported the functioning of nutritional status in alleviating environmental stress such as temperature and salinity (12, 13), there are limited studies on how to modulate osmotic pressure balance by nutritional regulation of cholesterol.

Cholesterol is a critical sterol serving as a precursor to physiologically active compounds in animals, including sex hormones, molting hormones, adrenal corticoids, bile acids, and vitamin D (14, 15). Additionally, cholesterol is a crucial component of membrane bilayers that regulate the stability and fluidity of the cellular membrane (16), which is vital to the osmoregulation of aquatic organisms. In other words, cholesterol is an indispensable component of the cellular membrane and can affect osmoregulatory capacity by changing its contents in the cellular membrane (17). Generally, cholesterol is usually free or chemically bound to fatty acids, and fatty acids, especially omega-3 long-chain polyunsaturated fatty acids (n-3 LC-PUFAs) can increase the unsaturation of the cell membrane, which is required to guarantee the regular operation of membrane proteins and ion transport (18). Moreover, because bony fish possess the ability to synthesize cholesterol (19), little attention has been paid on potential nutritional needs of bony fish for cholesterol, especially in marine fish. Previous studies on cholesterol nutrition in aquatic animals have been mainly focused on growth performance (15), cholesterol metabolism (20), interaction with phospholipids (21), and gonadal development in crustaceans (22), but detailed information on the impact of interaction between dietary cholesterol level and salinity on adaptation to low-salinity water environments of marine fish is scarce.

*Acanthopagrus schlegelii* is an important euryhaline marine fish species that is farmed commercially in various countries of Asia including China, Japan, and South Korea. It has been regarded as an excellent species for intensive aquaculture because it can resist diseases and tolerate a wide range of environmental conditions. (23–25). As an euryhaline fish, *A. schlegelii*is is widely farmed and cultured in coastal areas, bays, and natural harbors, where drastic changes in salinity inevitably occur. Besides, the production of seabream in China reached 122,449 tons in 2020 (26). Therefore, *A. schlegelii* could be a good model for studying physiological and biochemical responses to salinity environmental changes; in addition, it has practical significance to promote the cultivation of marine fish in low-salinity areas such as inland and estuaries. Hence, the objectives of this study were to investigate the physiological and biochemical responses of *A. schlegelii* to rearing in a low-salinity environment and explore whether these responses can be regulated by cholesterol nutrition strategies, and, finally, improve the adaptability of *A. schlegelii* to low-salinity environments.

## Materials and Methods

### Experimental Diets

Three isonitrogenous and isolipidic (39% protein and 19% lipid) experiment diets were formulated; the diets contained three levels of cholesterol (lamellate; Maclin Biochemical Technology Co., Ltd., Shanghai, China) and were named CH0.16 (supplemented with 0% cholesterol) CH1.0 (supplemented with 1% cholesterol), and CH1.6 (supplemented with 2% cholesterol) (Supplementary Table S1). Palmitic acid was added to the experimental diets to balance cholesterol content, ensuring the total amount of both is 2% in each diet. Fish meal, soybean protein concentrate, and soybean meal were used as protein sources; fish oil, soybean oil, and soybean lecithin were used as main lipid sources. All the used ingredients were purchased from Ningbo Tech-Bank Feed Co., Ltd. (Ningbo, China). Fatty acid compositions of the experimental diets are shown in Supplementary Table S2. The production process of the experimental diets was performed as previously described (27). Generally, all dry ingredients were ground with a hammer mill (H-28; South China University of Technology, Guangzhou, China). After passing through a 60-mesh sieve, the obtained ingredients as well as cholesterol (pulverized with a small mill) were weighed and thoroughly mixed in a Hobart-type blender, and cold-extruded pellets were produced (F-26, Machine factory of South China University of Technology) with pellet strands cut into uniform sizes (2-mm diameter pellets were prepared) (G-250; machine factory of South China University of Technology). The pellets were air-dried to ~10% moisture, sealed in vacuum-packed bags, and stored at −20°C until they were used in the feeding trial.

### Feeding Trial Conditions

*A. schlegelii* juveniles were obtained from a local commercial hatchery in Xiangshan Bay (salinity ranged from 23 to 25 psu in summer), Ningbo, China, and the feeding trial was carried out in a pilot base of Meishan Campus of Ningbo University (Ningbo, China). Prior to conducting the feeding trial, the fish were cultured in 100-L recirculation aquaculture system (RAS) aquaria for 2 weeks to get acclimated with the laboratory environment. Water was continuously purified with a series of filtration treatments including mechanical and bio-filter systems followed by ultraviolet (UV) treatment with controlled water temperature. During this period, half of the fish were, respectively, temporary reared in two salinity levels (normal, 23 psu and low, 5 psu) by feeding with the same commercial marine fish sinking pellet feed (1.5 mm sinking, ~45 % crude protein, and ~12 % crude lipid) obtained from Ningbo Tech-Bank Corp. The low-salinity water was obtained by diluting natural seawater with freshwater. *A. schlegelii* juveniles in the low salinity group were acclimated from 23- to 5-psu salinity by decreasing 2 psu/day for over ~9 days. The 5-psu water was prepared in a 1,000-L tank in advance, and then we pumped the diluted water into RAS of the low salinity group to maintain salinity. A total of 180 juveniles with an initial weight of 4.22 ± 0 g (90 obtained with each salinity) were normally and randomly allocated to 18 tanks (100 L) of two separate recirculating aquaculture system (RASs), which resulted in 10 fish per tank, with triplicate tanks for each of the six treatments. Fish reared in normal salinity water (~23 psu) were fed with the diets supplemented with 0, 1, and 2% cholesterol, respectively, termed NCH0.16, NCH1.0, and NCH1.6; while fish reared in low salinity water (~5 psu) were fed with the diets supplemented with 0, 1, and 2% cholesterol, respectively, termed LCH0.16, LCH1.0, and LCH1.6 (30 fish per treatment, 10 fish per tank). During the 4-week feeding trial period, the fish were fed at the same fixed rate (5–8% of wet initial body weight). In order to avoid fish stress response, weekly weighing was not carried out, so the feeding rate was increased by 1% of initial weight per week, and 40% of the aquarium water was renewed every 2 days to maintain water quality. Water quality parameters were measured weekly and mean values were as follows (mean ± SEM): salinity (low salinity, 5.11 ± 0.35 psu; normal salinity, 23.04 ± 0.0 psu), temperature 25.2 ± 1.04°C, dissolved oxygen 7.46 ± 0.91 mg/L, ammonia nitrogen 0.03 ± 0.11 mg/L, and pH 7.4 ± 0.34.

### Sample Collection

At the end of feeding trial, the fish were fasted for 24 h and then anesthetized with tricaine methane sulphonate (MS-222, 100 mg/L; Sinopharm Chemical Reagent Co., Ltd.) at 100 mg/L. All fish in each tank were weighed and counted to determine final body weight (FBW), weight gain (WG), specific growth rate (SGR), and survival rate (SR). Blood samples were collected with 2-ml syringes from the caudal vasculature of 6 fish per, and stored at 4°C for 24 h. Then, the blood samples were centrifuged at 956 × g for 10 min at 4°C to collect serum for analyzing biochemical indexes and enzyme activity. Then, the fish were drained of blood and continued to be used to collect gill and liver samples for analyzing biochemical indices and gene expression (pools of 3 fish per tank, *n* = 3), and further liver samples for fatty acid composition (pools of 3 fish per tank, *n* = 3), and stored at −80°C prior to analysis. Intestine samples were also collected and stored at −80°C until further analysis of total bile acid (TBA) content (pools of 3 fish per tank, *n* = 3).

### Proximate Composition Analysis

Lipid, protein, moisture, and ash contents of the were determined with standard AOAC methods (28). Moisture content was measured by drying the samples to constant weight at 105°C. Total lipid (crude lipid in the case of the feeds) was extracted with the ether extraction method using Soxtec System HT (Soxtec System HT6; Tecator, Sweden). Protein (crude protein in the case of the feeds) (N × 6.25) was determined according to the Dumas combustion method with a protein analyzer (FP-528; Leco, United States), and ash content was measured using a muffle furnace at 550°C for 8 h.

### Dietary Cholesterol Concentration Analysis

Dietary cholesterol concentrations in the experimental diets were analyzed with the gas chromatography (GC) method. Briefly, after extraction of lipid from 500-mg diets with 10 ml chloroform, we sampled 1 ml of the extracted lipid solution, dried under a pure nitrogen stream, and then the obtained residue was mixed with 1 ml diethyl ether. Finally, the samples were analyzed by gas chromatography (Shimadzu, Japan). This analysis was performed at the Institute of Analysis, Guangdong Academy of Sciences, China National Analytical Center (Guangzhou, China).

### Fatty Acid Composition Analysis

Fatty acids in the diets and liver were determined according to the methods of with some minor modifications (23). Briefly, 1 ml of methyl tricosanoate solution (23:0, 1 mg/ml) was added as internal standard to clean glass tubes, and the solvent evaporated using a Termovap sample concentrator (MIULAB, Hangzhou, China). Freeze-dried samples (diet samples ~120 mg and liver samples ~80 mg) were added to the tubes, and a 3-ml transmethylation solution (99 ml methanol:1 ml H_2_SO_4_:0.025 g butylated hydroxytoluene) was added. The mixtures were shaken for 10 min and then incubated for 4 h at 80°C in a water bath. The reactions were then cooled at room temperature before 1 ml of *n*-hexane was added and mixed thoroughly for 1 min, and before ultrapure water (1 ml) was added and the mixtures were shaken. After layer separation, the supernatant was filtered (0.22-μm ultrafiltration membrane; Millipore, MA, United States) into a clean vial and fatty acid methyl esters (FAMEs) were concentrated using a stream of nitrogen gas in a Termovap sample concentrator. The dried FAMEs were dissolved in 500-μl *n*-hexane and stored at −20°C prior to quantification by gas chromatography-mass spectrometry (GC-MS, Agilent 7890B-5977A; Agilent Technologies, CA, United States). The absolute quantity of each fatty acid was determined by calculating the ratio of the FAME peak area: internal standard (23:0) peak area with results obtained were then presented as content per mass of tissue (mg/g liver or diet, dry matter).

### Assay of Osmotic Pressure Regulation-Related Parameters

Serum samples were obtained as described above. Serum cortisol content was determined using an ELISA kit (Shanghai Qiaodu Biotechnology Co., Ltd., Shanghai, China). Similarly, serum sodium (Na^+^), potassium (K^+^), chloride (Cl^−^), magnesium (Mg^2+^), and calcium (Ca^2+^) concentrations, as well as Na^+^-K^+^-ATPase (NKA) activity in the gills, were determined according to the manufacturer's instructions using commercial kits (Nanjing Jiancheng Institute of Biological Engineering, Nanjing, China) with Multiskan Spectrum (Thermo Fisher Scientific, United States).

### Assay of Cholesterol Metabolism-Related Parameters

Samples of tissues (liver and intestine) were homogenized in nine volumes (w/v) of ice-cold physiological saline (0.89 %, w/v) before being centrifuged at 956 × g at 4°C for 10 min. Hepatic3-hydroxy-3-methylglutaryl coenzyme A reductase (HMGCR) concentration was determined using an ELISA kit (Shanghai Qiaodu Biotechnology Co., Ltd., Shanghai, China). Cholesterol (CHOL), high-density lipoprotein cholesterol (HDL-C), low-density lipoprotein cholesterol (LDL-C), and TBA contents were measured following the manufacturer's instructions using commercial kits (Nanjing Jiancheng Institute of Biological Engineering (Nanjing, China) with Multiskan Spectrum (Thermo Fisher Scientific, United States).

### Assay of Oxidative and Antioxidant Parameters

The activities of glutathione peroxidase (GSH-px) and superoxide dismutase (SOD), and total antioxidation capability (T-AOC), as well as the contents of glutathione (GSH) and malondialdehyde (MDA) were assayed using commercial kits purchased from Nanjing Jiancheng Bioengineering Institute (Nanjing, China) according to the manufacturer's instructions with the Multiskan Spectrum (Thermo Fisher Scientific, United States).

### Assay of Total RNA Extraction, and Reverse Transcription and Real-Time Quantitative PCR

Reverse-transcription quantitative PCR (qPCR) was performed to determine gene expression essentially as described previously but with some minor modifications (24). Extraction of total RNA from tissues of juvenile *A. schlegelii* was performed with the Trizol reagent according to instructions provided by the manufacturer (Vazyme Biotech Co., Ltd., Nanjing, China). The isolated RNA was subjected to analysis with a Nanodrop 2000 spectrophotometer (Thermo Fisher Scientific, United States) and gel electrophoresis (1.2% denatured agarose) to assess quantity and quality, respectively. PrimeScript™ RT Reagent Kit with gDNA Eraser (Perfect Real Time) was used to prepare cDNA from 1,000 ng of DNAase-treated RNA using the protocol supplied by the manufacturer (Vazyme). β*-actin* was used as a reference housekeeping gene after its stability across all the experimental samples was confirmed. For the RT-qPCR, specific primers were designed using the Primer Premier 5.0 software and cDNA sequences of the corresponding genes present in the database of the NCBI (Supplementary Table S3). Amplification and qPCR were carried out in a Lightcycler 96 instrument (Roche, Switzerland). Specifically, qPCR was carried out in reaction volumes of 20 μl that contained 1 μl of each primer, 10 μl of double-concentrated SYBR Green I Master (Vazyme), 2 μl of 1/10 diluted cDNA, and 6 μl diethyl pyrocarbonate-treated water. The real time PCR conditions were as follows: 95°C for 2 min, 45 cycles of 95°C for 10 s, 58°C for 10 s, and, finally, 72°C for 20 s. Standard calibration curves were prepared from six individual dilution concentration gradients of cDNA samples, and amplification efficiency was determined using the E = 10^(−1/*Slope*)^-1 equation, which showed that efficiencies were approximately equal (range 90 to 110%) for all the genes. Expression levels of target genes were calculated using the 2^−Δ*ΔCt*^ method (29), and gene expression data were presented relative to the expression of the NCH0.16 (reference) group. The relative gene expression results were expressed as mean normalized ratios corresponding to the ratio between the copy numbers of target genes and copy numbers of the reference gene, β*-actin*.

### Statistical Analysis

The results are presented as means ± SEM (number of replicates as indicated). One-way analysis of variance (ANOVA) was performed for different dietary cholesterol levels under both water salinity. Prior to the one-way ANOVA, homogeneity of variances was checked by Levene's test. Following the significant ANOVA, Tukey's range test was conducted to determine significant differences between means, with a significance level of *P* < 0.05 applied. Independent-samples Student's *t*-test was performed to evaluate the significant difference between the low salinity and normal salinity groups with same dietary cholesterol level. In addition, a two-way ANOVA was performed to determine the interaction between dietary cholesterol levels (0, 1, and 2%) and water salinity (5 and 23 psu) (the results are shown in Supplementary File). The results were considered to be significant and highly significant at *P* < 0.05 and *P* < 0.01, respectively. All the statistical analyses were performed using the SPSS software (IBM SPSS Statistics 20, United States).

A principal component analysis (PCA) of fatty acid composition in the liver was carried out with the SIMCA-P+ software (Version 11.0.0.0; Umetrics AB, Malmo, Sweden) to understand the communalities and discrepancies of effects of dietary cholesterol level and water salinity on *A. schlegelii*. The hierarchical cluster analysis (HCA) was performed to analyze relationships between the different sample fatty acid profiles utilizing Pearson correlation and average clustering algorithm subsequent to log2 transformation. Heat maps were used to visualize the complex fatty acid data sets organized as Pearson correlation matrices. The free online program Image GP (http://www.ehbio.com/ImageGP/index.php/) was used for both the HCA and heat map visualization.

## Results

### Growth Performance

The impacts of dietary cholesterol level and water salinity on growth performance of *A. schlegelii* juveniles are presented in Table 1. The results of the two-way ANOVA showed that the final body weight (FBW), weight gain (WG), and specific growth rate (SGR) were significantly impacted by dietary cholesterol level and water salinity (*P* < 0.01), but that no significant interactions were found between dietary cholesterol level and water salinity (*P* > 0.05). Additionally, the *t*-test analysis indicated that when fed with the same dietary cholesterol level, fish reared in low salinity water (LCH1.0 and LCH1.6 treatments) had markedly improved growth performance (FBW, WG, and SGR) compared with fish reared in normal salinity (NCH1.0 and NCH1.6 treatments). Moreover, the one-way ANOVA showed that the fish fed with the diet containing 1% cholesterol (NCH1.0 and LCH1.0 treatments) had significantly higher growth performance (FBW, WG, and SGR) than the dietary CH0.16 group (NCH0.16 and LCH0.16 treatments) in both water salinity. With regard to survival rate, no significant differences were found among the treatments (*P* > 0.05).

**Table 1:** Growth performance of *Acanthopagrusschlegelii* fed experimental diet reared at different water salinity for 4 weeks.

**Items**	**Dietary treatments in normal water salinity**			**Dietary treatments in low water salinity**		
	**NCH0.16**	**NCH1.0**	**NCH1.6**	**LCH0.16**	**LCH1.0**	**LCH1.6**
IBW (g)	4.22 ± 0.03	4.21 ± 0.09	4.23 ± 0.03	4.22 ± 0.03	4.21 ± 0.09	4.22 ± 0.09
FBW (g)	9.72 ± 0.38^A^	11.34 ± 0.46^B^	10.59 ± 0.19^AB^	10.90 ± 0.43^a^	14.43 ± 0.98^b#^	13.20 ± 0.74^ab#^
WG (%)	130.15 ± 9.03^A^	168.97 ± 11.42^B^	150.62 ± 4.77^AB^	157.91 ± 10.34^a^	241.99 ± 22.89^b#^	213.07 ± 17.16^ab#^
SGR (%/ d)	2.97 ± 0.14^A^	3.53 ± 0.15^B^	3.28 ± 0.07^AB^	3.38 ± 0.48^a^	4.43 ± 0.26^b#^	4.06 ± 0.20^ab#^
SR (%)	100.00 ± 0.00	90.00 ± 5.77	100.00 ± 0.00	93.33 ± 3.33	96.67 ± 3.33	93.33 ± 3.33
**Items**	***P*** **-values (two-way ANOVA)**					
	**Cholesterol**		**Salinity**		**Cholesterol** *salinity	
FBW (g)	<0.01		<0.01		0.28	
WG (%)	<0.01		<0.01		0.27	
SGR (%/d)	<0.01		<0.01		0.28	
SR (%)	0.53		0.43		0.11	

*Values are presented as the means ± SEM of three replicates (n = 3). P-values of influences of dietary cholesterol level, water salinity and their interaction on the relevant parameters are presented. “AB” representing significant (P < 0.05) difference between dietary cholesterol levels in normal water salinity (23 psu) and “ab” representing significant (P < 0.05) difference between dietary cholesterol levels in low water salinity (5 psu) by performing one-way ANOVA. “#” representing significant (P < 0.05) difference between water salinity with same dietary cholesterol level by performing t-test. IBW, Initial body weight; FBW, Final body weight; Weight gain (WG, %) = 100× (final body weight-initial body weight)/finial body weight; Specific growth ratio (SGR), SGR (%/ d) = 100× [Ln (final body weight) – Ln (initial body weight)]/28 days. Survival rate (SR), SR (%) = 100 (final fish number/initial fish number)*.

### Key Parameters of Osmoregulation

**Figure 1:**
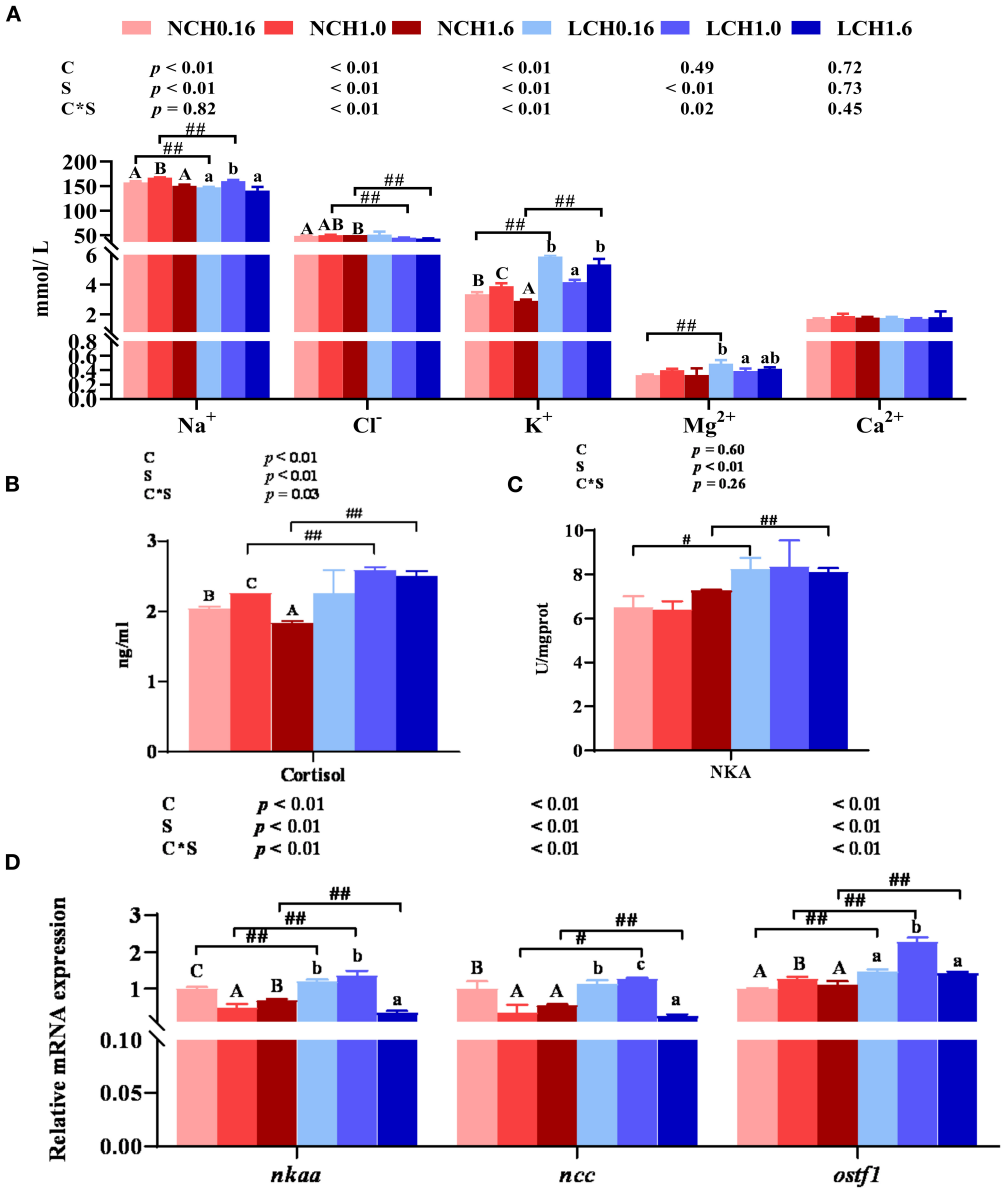
Effects of dietary cholesterol level and water salinity on osmoregulation ability of juvenile black seabream (*Acanthopagrus schlegelii*). Concentrations of ions inserum **(A)**, cortisol concentrations inserum **(B)**, Na^+^ / K^+^-ATPase activitiesin gill **(C)**, andrelative gene expression levels involved in osmoregulation in gill **(D)**. The group of NCH 0.16 was set as the reference group, and the mRNA expression levels of target genes were normalized relative to the expression of β*-actin*. Values are presented as the means (*n* = 3), with standard errors represented by vertical bars. Two-way ANOVA *P*-values are shown in each panel, with “C” representing effects of dietary cholesterol levels, “S” representing effects of salinity, and “C*S” representing interaction between dietary cholesterol levels and salinity. “ABC” representing significant (*P* < 0.05) difference between dietary cholesterol levels in normal water salinity (23 psu) and “abc” representing significant (*P* < 0.05) difference between dietary cholesterol levels in low water salinity (5 psu) by performing one-way ANOVA. “#” representing significant (*P* < 0.05) difference between water salinity with same dietary cholesterol level and “##” representing highly significant (*P* < 0.01) difference between water salinity with same dietary cholesterol level by performing *t*-test. Ca^2+^, calcium; Mg^2+^, magnesium; Na^+^, sodium, Cl^−^, chlorine; K^+^, potassium; *ostf1*, osmotic stress transcription factor 1; *ncc*, Na^+^-Cl^−^ cotransporter; *nka*α, Na^+^/K^+^-ATPase.

The effects of dietary cholesterol level and water salinity on key parameters of osmoregulation are shown in Figure 1. The two-way ANOVA showed that water salinity significantly influenced all the measured parameters related to osmoregulation, except for Ca^2+^ content in the serum, and that dietary cholesterol level dramatically influenced the concentrations of Na^+^, K^+^, and cortisolin serum as well as the relative expression levels of Na^+^/K^+^-ATPase (*nka*α), Na^+^-Cl^−^ co-transporter (*ncc*), and osmotic stress transcription factor 1 (*ostf1*) in the gills (*P* < 0.05). Significant interactions between dietary cholesterol level and water salinity were reflected on the concentration of Cl^−^, K^+^, Mg^2+^ and cortisol in serum and relative expression levels of *nka*α, *ncc*, and *ostf1* in the gills (*P* < 0.05). Specifically, in the serum, when compared to those reared in normal salinity, fish reared in low water salinity had significantly increased contents of Mg^2+^ (LCH0.16 group), K^+^(LCH0.16 and LCH1.0 groups), and cortisol (LCH0.16, LCH1.0, and LCH1.6 groups) (*P* < 0.01) but decreased contents of Na^+^(LCH0.16 and LCH1.0) and Cl^−^(LCH1.0 and LCH1.6 groups) (*P* < 0.01) (Figures 1A,B). Fish fed with dietary CH1.0 had markedly enhanced concentrations of Na^+^ (NCH1.0 and LCH1.0 groups) and K^+^ (NCH1.0 group), but decreased K^+^ (LCH1.0 group) and Mg^2+^ (LCH1.0 groups) contents under low salinity compared to fish fed with cholesterol-free diet (NCH0.16 and LCH0.16 groups) (Figure 1A) (*P* < 0.05). Diet CH1.0 (LCH1.0) led to significantly increased cortisol content in low salinity level, while cortisol content was significantly decreased with decrease of dietary cholesterol level in normal salinity (*P* < 0.01) (Figure 1B). Fish cultured in low salinity (LCH0.16 and LCH1.6 groups) showed significantly higher NKA activity than fish cultured in normal salinity (NCH0.16 and NCH1.6 groups) (*P* < 0.05) (Figure 1C). Fish fed with the diet containing 1% cholesterol (LCH1.0 group) had markedly upregulated gene expression levels of *ncc* and *ostf1* when cultured in low salinity (*P* < 0.01) (Figure 1D). However, the content of Ca^2+^ in the serum did not show any statistical differences among the treatments (*P* > 0.05) (Figure 1A).

### Key Parameters of Cholesterol Metabolism

**Figure 2:**
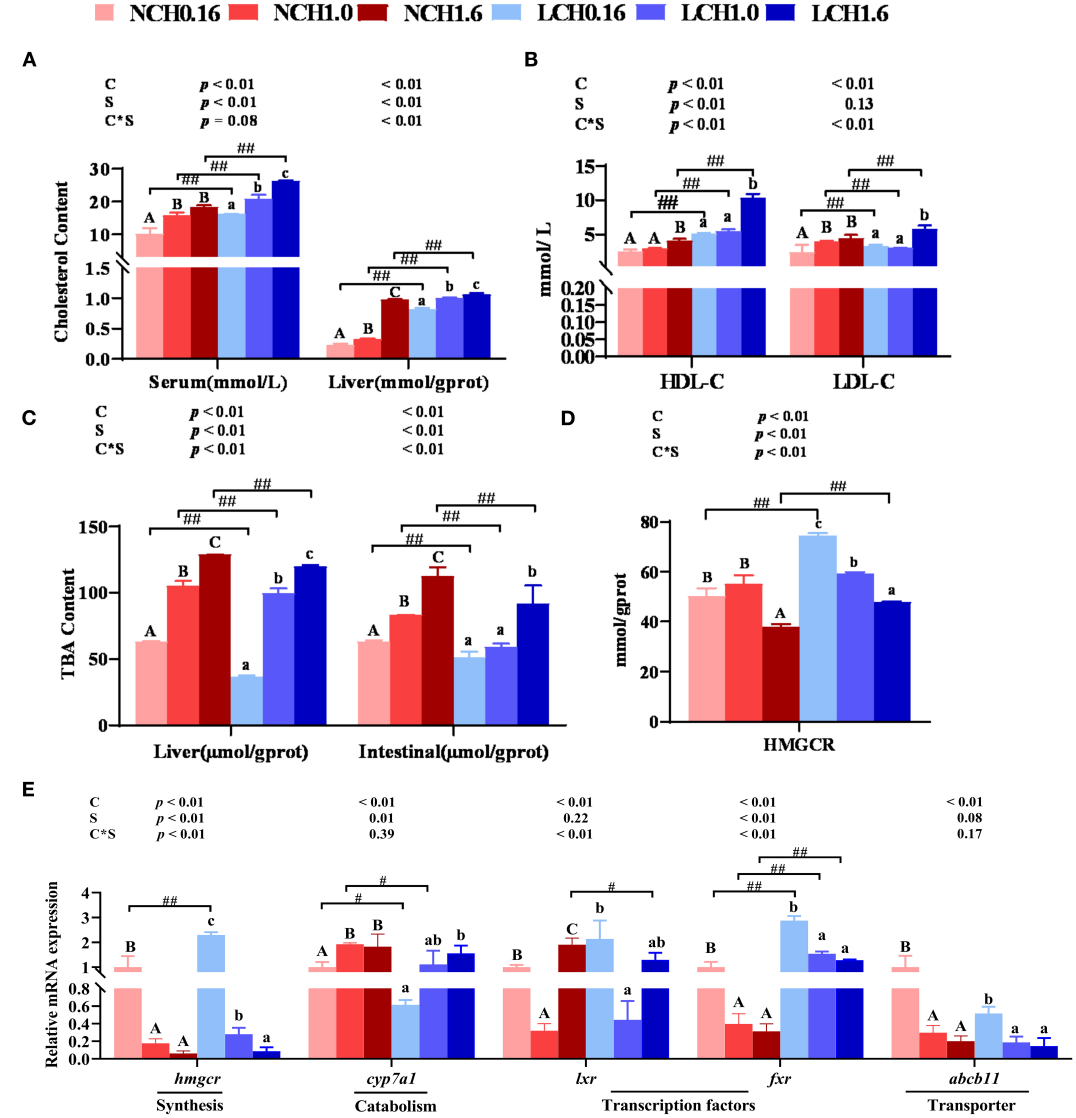
. Effects of dietary cholesterol level and water salinity on cholesterol metabolism of juvenile black seabream (*Acanthopagrusschlegelii*). Contents of cholesterol in serum and liver **(A)**, content of total bile acid in serum and liver **(B)**, Hmgcr enzyme activities in liver **(C)**, concentrations of cholesterol transport in serum **(D)**, and relative genes expression involved in cholesterol metabolism in liver **(E)**. The group of NCH0.16 was set as the reference group, and the mRNA expression levels of target genes were normalized relative to the expression of β-actin. Values are presented as the means (*n* = 3), with standard errors represented by vertical bars. Two-way ANOVA *P*-values are shown in each panel, with “C” representing effects of dietary cholesterol levels, “S” representing effects of salinity, and “C*S” representing the interaction between dietary cholesterol levels and salinity. “ABC” representing significant (*P* < 0.05) difference between dietary cholesterol levels in normal water salinity (23 psu) and “abc” representing significant (*P* < 0.05) difference between dietary cholesterol levels in low water salinity (5 psu) by performing one-way ANOVA. “#” representing significant (*P* < 0.05) difference between water salinity with same dietary cholesterol level and “##” representing highly significant (*P* < 0.01) difference between water salinity with same dietary cholesterol level by performing *t-*test. TBA, total bile acid; HDL-C, high-density lipoprotein cholesterol; LDL-C, low-density lipoprotein cholesterol; hmgcr, 3-Hydroxy-3-Methylglutaryl-CoA Reductase; cyp7a1, cholesterol 7α-hydroxylase; lxr, liver x receptor; fxr, farnesoid X receptor; abcg5, ATP bind cassrtte transport g5; abcb11, ATP bind cassrtte transport b11.

The two-way ANOVA showed that most of the key parameters of cholesterol metabolism measured in this study were significantly affected by both dietary cholesterol level and water salinity (*P* < 0.05) (Figure 2). Extremely significant interactions between dietary cholesterol level and water salinity were observed in all the tested parameters except for cholesterol 7α-hydroxylase (*cyp7a1*) and ATP-binding cassette transport b11 (*abcb11*) relative expression levels in the liver (*P* < 0.01). In detail, low salinity (LCH0.16, LCH1.0, and LCH1.6 treatments) dramatically increased the contents of cholesterol in the serum and liver as well as serum HDL-C and LDL-C contents compared to the normal salinity and, as shown in Figures 2A,B, when cultured in the same salinity, these values were increased with increase of dietary cholesterol level (both water salinity) (*P* < 0.01). Fish fed with the diet supplemented with cholesterol had significantly increased content of TBA in the liver and intestine, and showed a significant dose effect in both two-water salinity (*P* < 0.01). Furthermore, low salinity (LCH0.16, LCH1.0, and LCH1.6 treatments) markedly reduced TBA content in the tested tissues (*P* < 0.01) (Figure 2C). Besides, HMGCR activity in the liver was dramatically increased by low salinity (LCH0.16 and LCH1.6 treatments); however, HMGCR activity was decreased with increase of dietary cholesterol levels in fish fed in both water salinity (*P* < 0.01) (Figure 2D). With regard to gene expression levels related to cholesterol metabolism, the tested genes were mainly affected by dietary cholesterol level and, to a lesser extent, water salinity (Figure 2E). The specific data showed that low salinity led to significantly increased hepatic gene expression levels of *hmcgr* (LCH0.16 group) and *fxr* (LCH0.16, LCH1.0, and LCH1.6) than the normal salinity groups accordingly (*P* < 0.01). Additionally, fish reared in the same water salinity and fed with the diet supplemented with cholesterol exhibited significantly lower relative expression levels of *hmcgr, fxr*, and *abcb11* than the cholesterol-free diet groups (*P* < 0.05). However, contrary results were observed in hepatic *cyp7a1* relative expression level, as it was dramatically upregulated by dietary cholesterol in both water salinity (*P* < 0.05). Low salinity (LCH0.16 and LCH1.0 treatments) significantly decreased the gene expression level of *cyp7a1* in the liver compared to the normal salinity groups (*P* < 0.05). Additionally, hepatic *lxr* expression level varied with dietary cholesterol levels (*P* < 0.05).

### Fatty Acid Composition and LC-PUFA Biosynthesis Pathway Markers in the Liver

**Figure 3:**
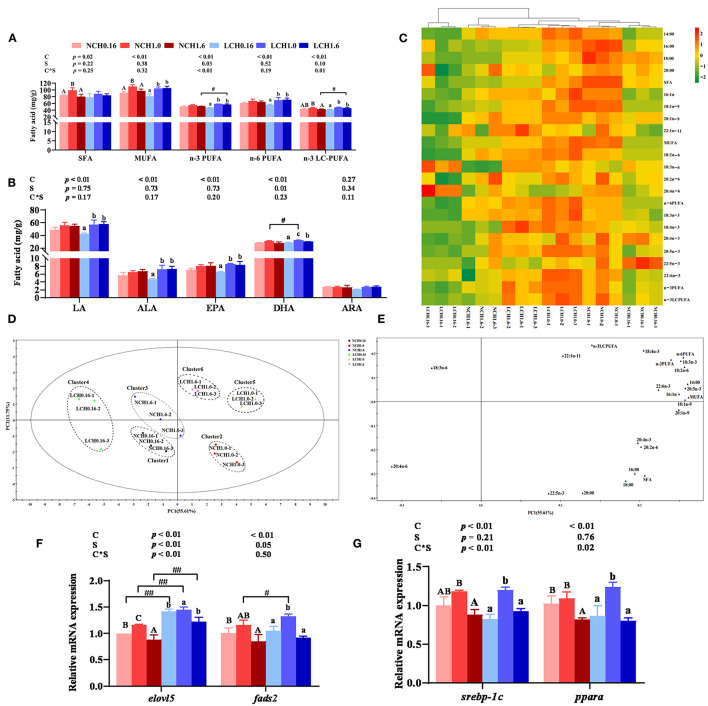
Effects of dietary cholesterol level and water salinity on LC-PUFA biosysnthesis and hepatic fatty acid profiles of juvenile black seabream (*Acanthopagrus schlegelii*). Main fatty acid profile in liver **(A)** and **(B)**, hierarchical cluster analysis (HCA) and heat map visualization of the main fatty acid composition in liver **(C)**, principal component analysis (PCA) score plot **(D)** and loading plot **(E)** results of fatty acids in liver; hepatic relative gene expression levels involved in LC-PUFA biosynthetic pathway **(F)** and hepatic relative gene expression levels of lipid metabolism-related transcription factors. Cluster 1 represented as the NCH0.16 diet group, Cluster 2 represented as the NCH1.0 diet group, Cluster3 represented as the NCH1.6 diet group, Cluster 4 represented as the LCH0.16 diet group, Cluster 5 represented as the LCH1.0 diet group and Cluster 6 represented as the LCH1.6 diet group. Hierarchical cluster analysis (HCA) and heat map visualization (c) for Pearson's correlation analysis of the correlation matrix of the fatty acid composition. The group of NCH0.16 was set as the reference group, and the mRNA expression levels of target genes were normalized relative to the expression of β*-actin*. Values are means (*n* = 3) with standard errors represented by vertical bars. Two-way ANOVA *P*-values are shown in each panel, with “C” representing effects of dietary cholesterol levels, “S” representing effects of salinity, and “C*S” representing interaction between dietary cholesterol levels and salinity. “ABC” representing significant (*P* < 0.05) difference between dietary cholesterol levels in normal water salinity (23 psu) and “abc” representing significant (*P* < 0.05) difference between dietary cholesterol levels in low water salinity (5 psu) by performing one-way ANOVA. “#” representing significant (*P* < 0.05) difference between water salinity with same dietary cholesterol level and “##” representing highly significant (*P* < 0.01) difference between water salinity with same dietary cholesterol level by performing *t*-test. ARA, 20:4n-6; DHA, 22:6n-3; EPA, 20:5n-3; DHA/EPA, 22:6n-3/20:5n-3; LC-PUFA, long-chain polyunsaturated fatty acid (C20-24); MUFA, monounsaturated fatty acids; PUFA, polyunsaturated fatty acid; SFA, saturated fatty acids; *elovl5*, elongase of very long chain fatty acids 5; *fads2*, fatty acyl desaturase 2; *ppar*α, peroxisomeproliferator-activated receptor α; *srebp-1c*, sterol regulatory element-binding protein 1c.

The effects of dietary cholesterol level and water salinity on the fatty acid profile of the liver are presented in Figure 3, while the complete fatty acid compositions are shown in Supplementary Table S4. The two-way ANOVA indicated that the amounts of most fatty acids in the liver were markedly influenced by dietary cholesterol level and, to a lesser extent, water salinity (*P* < 0.05). Significant interactions between dietary cholesterol level and water salinity were found in total n-3 poly unsaturated fatty acid (n-3 PUFAs) and n-3 long-chain PUFA (n-3 LC-PUFA) contents (Figure 3A). The contents of total saturated fatty acids (SFAs), monounsaturated fatty acids (MUFAs), n-6 PUFAs (including linoleic acid and LA), n-3 PUFAs (including linolenic acid and ALA), and n-3 LC-PUFAs (including eicosapentaenoic acid, EPA, and docosahexaenoic acid, DHA) were significantly affected by dietary cholesterol level (*P* < 0.05) (Figures 3A,B). Accordingly, fish fed with the diets supplemented with cholesterol significantly increased most of the presented fatty acids in Figures 3A,B compared to the CH0.16 groups in the same water salinity, except for arachidonic acid (ARA) (*P* < 0.05). Low salinity slightly increased the DHA (LCH1.0 group), total n-3 PUFA (LCH1.6) and n-3 LC-PUFA (LCH1.6) concentrations in the liver of the fish compared to normal salinity (*P* < 0.05).

**Figure 4:**
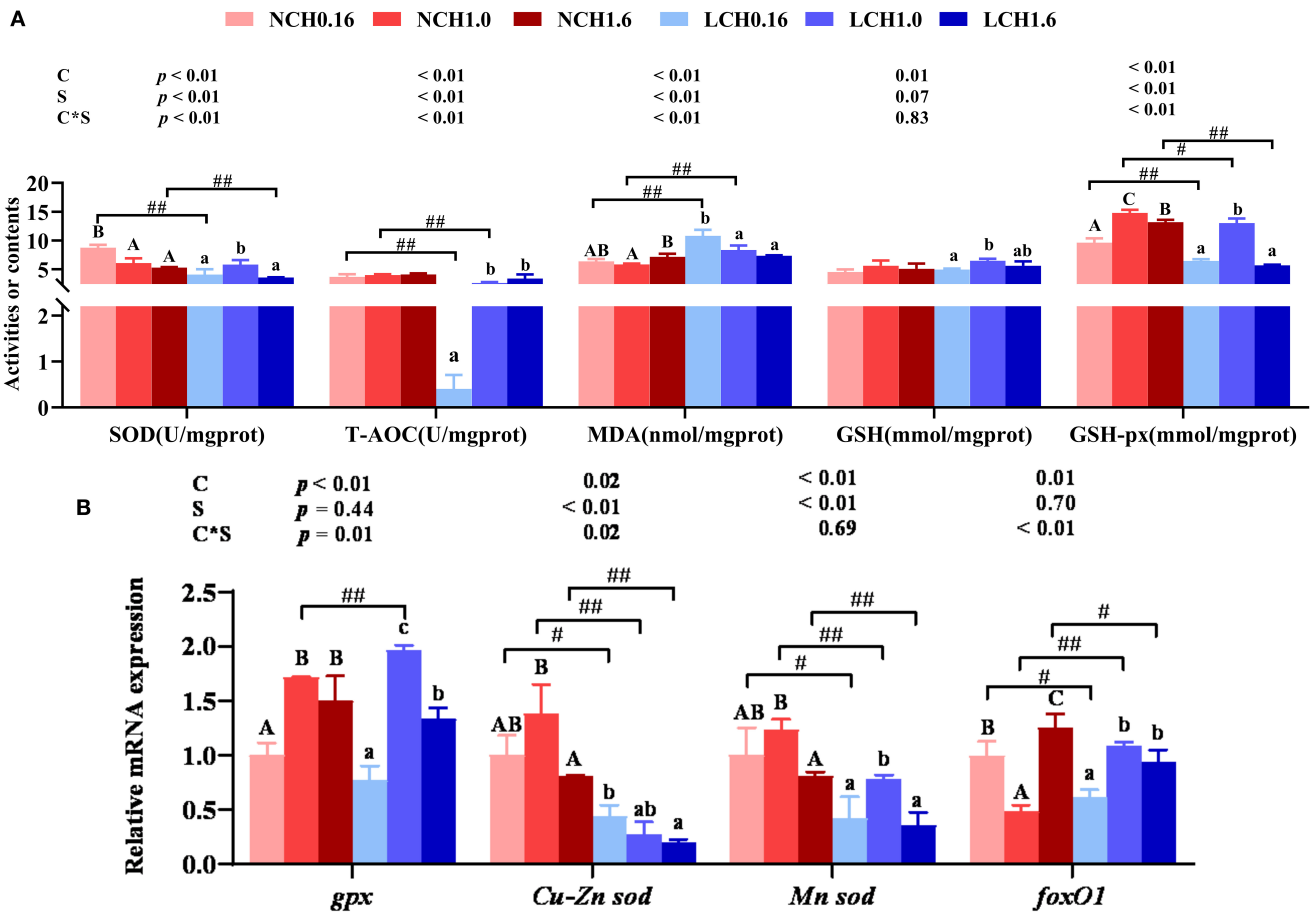
Effects of dietary cholesterol level and water salinity on oxidative stress and antioxidant capacityin liver of juvenile *Acanthopagrusschlegelii*. oxidation and antioxidant parameters in liver **(A)** and relative gene expression levels involved in antioxidant capacity in liver **(B)**. The group of NCH0.16 was set as the reference group, and the mRNA expression levels of target genes were normalized relative to the expression of β*-actin*. Values are presented as the means (*n* = 3), with standard errors represented by vertical bars. Two-way ANOVA *P*-values are shown in each panel, with “C” representing effects of dietary cholesterol levels, “S” representing effects of salinity, and “C*S” representing interaction between dietary cholesterol levels and salinity. “ABC” representing significant (*P* < 0.05) difference between dietary cholesterol levels in normal water salinity (23 psu) and “abc” representing significant (*P* < 0.05) difference between dietary cholesterol levels in low water salinity (5 psu) by performing one-way ANOVA. “#” representing significant (*P* < 0.05) difference between water salinity with same dietary cholesterol level and “##” representing highly significant (*P* < 0 .01) difference between water salinity with same dietary cholesterol level by performing *t*-test. GSH, glutathione; GSH-px, glutathione peroxidase; SOD, superoxidedismutase; T-AOC, total antioxidation capability; MDA, malonaldehyde; GSH, glutathione; GSH-px, glutathione peroxidase; *gpx*, glutathione peroxidase;*Cu-zn sod*, Cu-Zn superoxide dismutase; *Mn-sod*, Mn superoxidedismutase*; foxO1*, forkhead O1.

**Figure 5:**
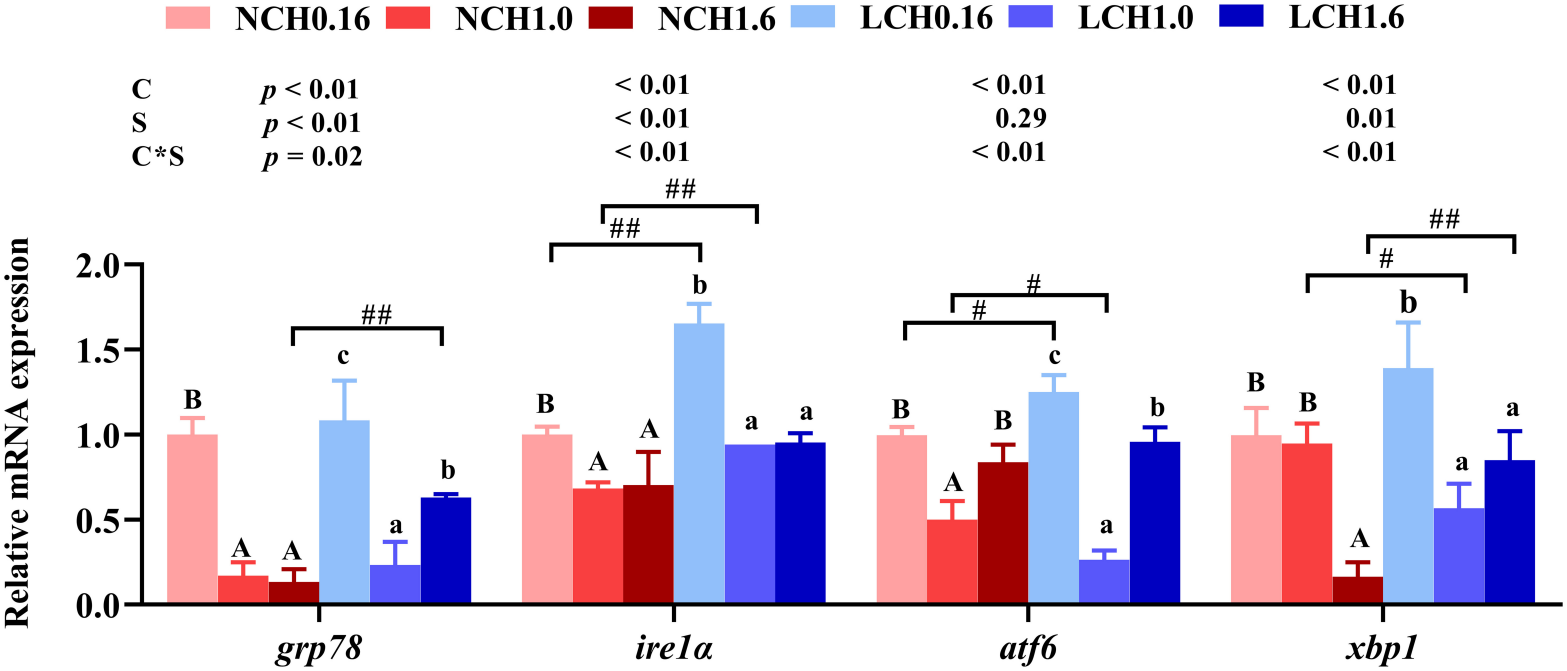
Effects of different dietary cholesterol level and water salinity on the relative mRNA expression involved in endoplasmic reticulum stress in juvenile black seabream (*Acanthopagrus schlegelii*). The group of NCH0.16 was set as the reference group, and the mRNA expression levels of target genes were normalized relative to the expression of β*-actin*. Values are means (*n* = 3) with standard errors represented by vertical bars. Two-way ANOVA *P*-values are shown in each panel, with “C” representing effects of dietary cholesterol levels, “S” representing effects of salinity, and “C*S” representing interaction between dietary cholesterol levels and salinity. “ABC” representing significant (*P* < 0.05) difference between dietary cholesterol levels in normal water salinity (23 psu) and “abc” representing significant (*P* < 0.05) difference between dietary cholesterol levels in low water salinity (5 psu) by performing one-way ANOVA. “#” representing significant (*P* < 0.05) difference between water salinity with same dietary cholesterol level and “##” representing highly significant ( *P* < 0.01) difference between water salinity with same dietary cholesterol level by performing *t*-test.*grp78*, glucose regulated protein 78; *ire1*α, inositol requiring enzyme 1α; *atf6*, activating transcription factor 6; *xbp1*, X-box binding protein 1.

**Figure 6:**
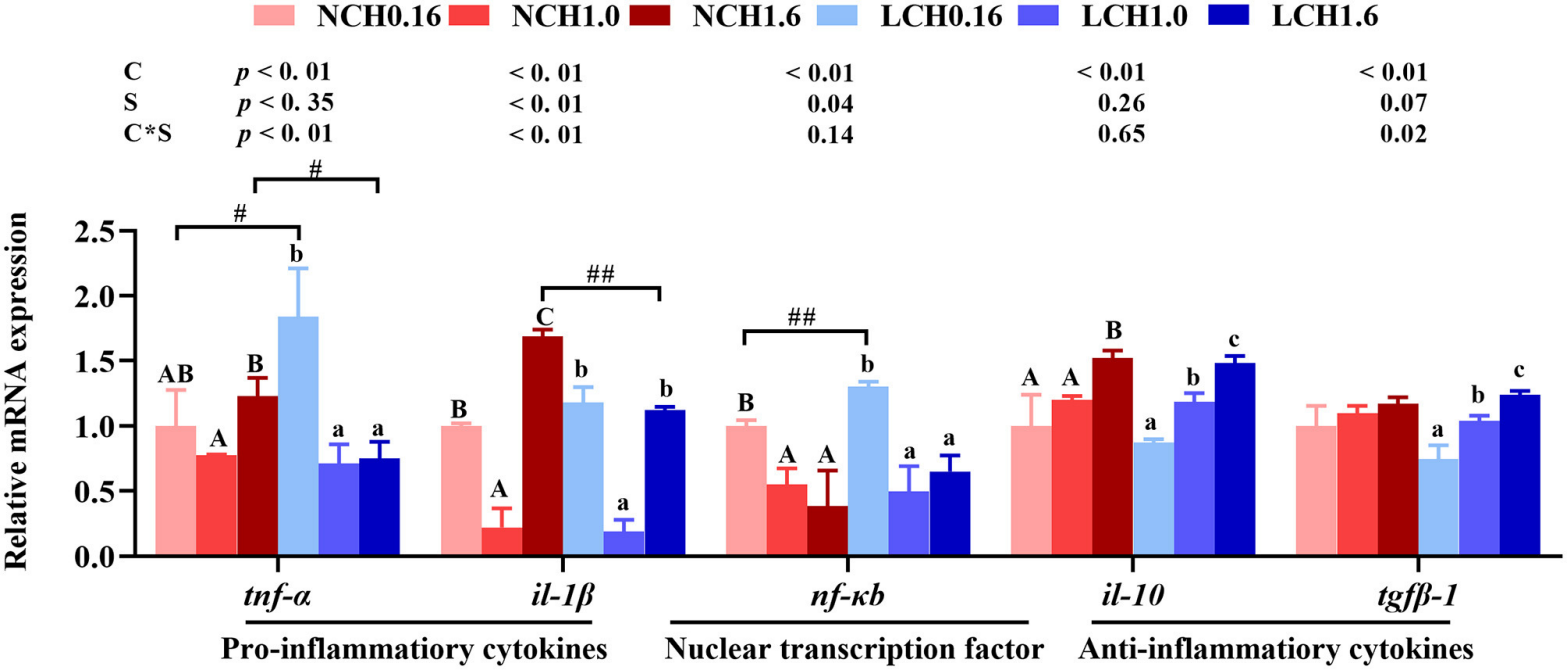
Effects of dietary cholesterol level and water salinity on hepatic relative mRNA expression involved in inflammatory response in liver of juvenile *Acanthopagrusschlegelii*. The group of NCH0.16 was set as the reference group, and the mRNA expression levels of target genes were normalized relative to the expression of β*-actin*. Values are means (*n* = 3) with standard errors represented by vertical bars. Two-way ANOVA *P*-values are shown in each panel, with “C” representing effects of dietary cholesterol levels, “S” representing effects of salinity, and “C*D” representing interaction between dietary cholesterol levels and salinity. “ABC” representing significant (*P* < 0.05) difference between dietary cholesterol levels in normal water salinity (23 psu) and “abc” representing significant (*P* < 0.05) difference between dietary cholesterol levels in low water salinity (5 psu) by performing one-way ANOVA. “#” representing significant (*P* < 0.05) difference between water salinity with same dietary cholesterol level and “##” representing highly significant (*P* < 0.01) difference between water salinity with same dietary cholesterol level by performing *t*-test. *il-10*, interleukin-10; *il-1*β, interleukin-1β; *nf-*κ*b*, nuclear factor kappa B; *tgf*β*-1*, transforming growth factor β-1; *tnf-*α, tumor necrosis factor α.

A heat map for the Pearson's correlation analysis of the correlation matrix of main detection indexes is presented in Figure 3C. Heat map visualization was conducted to present the macroscopic effects of dietary cholesterol level and water salinity on fatty acids composition of the liver. All the data were normalized, with red color representing higher values and green color representing lower values. The results clearly indicated that higher values of total SFA, MUFA, n-3PUFA, n-6 PUFA, n-3LC-PUFA, LA, ALA, EPA, and DHA were observed in fish fed with the CH1.0 diet and reared in both water salinity compared to the other groups, and that two groups (LCH1.0 and NCH1.0 groups) clustered close to each other. Principal component analysis (PCA) score plot and loading plot of fatty acid composition of the liver are presented in Figures 3D,E, respectively. The first two principal components (PCs) were 67.36% of the variation (55.61 and 11.75% of the total variance, respectively). As shown in Figure 3E, all the replicates of three groups were divided into six clusters: cluster 1 (NCH0.16), cluster 2 (NCH1.0 group), cluster 3 (NCH1.6 group), cluster 4 (LCH0.16 group), cluster 5 (LCH1.0 group), and cluster 6 (LCH1.6 group), and the six clusters were intuitively separated. The PCA loading plot demonstrated the distribution of hepatic fatty acid data affected by dietary cholesterol level and water salinity. Combining the information in Figures 3D,E, n-6PUFA, n-3 PUFA, n-3 LC-PUFA, EPA (20: 5n-3), and DHA (22:6n-3) were at the upper right of PC1, and were highly correlated with the LCH1.0 group.

The two-way ANOVA indicated that the relative expression levels of key genes of the LC-PUFA biosynthesis pathway in the liver were significantly affected by both dietary cholesterol level and water salinity (Figure 3F). Significant interactions between dietary cholesterol level and water salinity were observed in the expression levels of elongase of very long-chain fatty acids 5 (*elovl5*) (*P* < 0.01). The expression level of fatty acyl desaturase 2 (*fads2*) decreased with increase of dietary cholesterol level in both water salinity (*P* < 0.05). Fish fed diet with CH1.0 (NCH1.0 and LCH1.0 groups) had significantly upregulated expression of *elovl5* compared to the other groups in both water salinity (*P* < 0.05). Low salinity markedly increased the expression levels of *fads2* (LCH1.0 group) and *elovl5* (LCH0.16, LCH1.0, and LCH1.6) when fish were fed with the same diet (*P* < 0.05). Furthermore, relative gene expression levels of lipid metabolism-related transcription factors peroxisome proliferator-activated receptor α (*ppar*α) and sterol regulatory element-binding protein 1c (*srebp-1c*) in the liver are presented in Figure 3G. *Srebp-1c* and *ppar*α showed the same pattern, when fish reared at the same water salinity, the expression levels of these two genes were all markedly up-regulated by dietary CH1.0 compared to other treatments.

### Oxidation and Antioxidant Parameters in the Liver

The oxidative and antioxidant parameters in liver are presented in Figure 4. Two-way ANOVA showed that most of the measured parameters were dramatically affected by both dietary cholesterol level and water salinity, as well as interactions between dietary cholesterol level and water salinity (*P* < 0.05). Concretely, the *t*-test analysis showed that low salinity significantly decreased the activities of SOD (LCH0.16 and LCH1.6 groups) T-AOC (LCH0.16 and LCH1.6 groups) and GSH-px (LCH0.16, LCH1.0, and LCH1.6 groups) in liver (*P* < 0.01). On the contrary, low salinity markedly decreased the MDA content of the liver regardless of the dietary cholesterol level (*P* < 0.01) (Figure 4A) In addition, the relative expression levels of Cu-Zn superoxide dismutase (*Cu-Zn sod*) (LCH0.16, LCH1.0, and LCH1.6 groups), Mn superoxide dismutase (*Mn sod*) (LCH0.16, LCH1.0, and LCH1.6 groups) and forkhead O1 (*foxO1*) (LCH0.16 and LCH1.6 groups) in fish reared at low salinity reduced significantly, but dietary CH1.0 (LCH1.0 treatment) dramatically up-regulated *gpx* and *foxO1* expression levels compared to the NCH1.0 group (*P* < 0.05)(Figure 4A). One-way ANOVA indicated that fish cultured at low salinity fed with dietary CH1.0 significantly improved SOD, T-AOC, and GSH-px activities as well as GSH content compared to LCH0.16 group (*P* < 0.05), while MDA content was decreased with the increase of dietary cholesterol level (*P* < 0.05). Furthermore, CH1.0-fed fish displayed markedly higher expression levels of *gpx* (NCH1.0 and LCH1.0 treatments), *Cu-Zn sod* (NCH1.0), *Mn sod* (LCH1.0 treatment) and *foxO1* (LCH1.0 treatment) in liver compared to fish fed CH0.16 diet with the same water salinity (*P* < 0.05).

### Endoplasmic Reticulum Stress Markers in Liver

The mRNA expression levels of genes related to endoplasmic reticulum stress including glucose regulated protein 78 (*grp78*), inositol requiring enzyme-1 α (ire1α), activating transcription factor 6 (*atf6*) and X-box binding protein 1(*xbp1*) in liver were determined (Figure 5). The results of two-way ANOVA showed that *grp78, ire1*α, *atf6*, and *xbp1* expression levels were significantly affected by the dietary cholesterol level and water salinity (*P* < 0.01), except for *atf6* which was not significantly influenced by water salinity (*P* > 0.0*5*). Likewise, highly significant interaction between dietary cholesterol level and water salinity were recorded in all genes (*grp78, ire1*α, *atf6*, and *xbp1*) of ERS measured in the present study (*P* < 0.01). Specifically, *t*-test analysis indicated that when fed the same dietary cholesterol level, low salinity had dramatically higher *grp78* (LCH1.6 group), *ire1*α (LCH0.16 and LCH1.0 groups) and *atf6* (LCH0.16 group) expression level than those in fish reared at normal salinity (*P* < 0.05). Moreover, one-way ANOVA revealed that when the fish were cultured at the same water salinity, dietary CH1.0 markedly suppressed all the expression levels of ERS relevant genes compared to the fish fed dietary CH0.16 (*P* < 0.05), except for *xbp1* in normal water salinity (*P* > 0.05).(Figure 5).

### Inflammation Markers in Liver

The relative expression levels of genes of the inflammatory response including nuclear transcription factor kappa B (*nf-kb*), pro-inflammatory cytokines interleukin-1β (*il-1*β) and tumor necrosis factor α (*tnf-*α) as well as anti-inflammatory cytokines transforming growth factor β-1 (*tgf*β*-1*) and interleukin-10 (*il-10*) in liver and intestine are shown in Figure 6. Two-way ANOVA showed that gene markers of inflammatory response (including *nf-kb, il-1*β, *tnf-*α, *tgf*β*-1*, and *il-10*) were significantly affected by dietary cholesterol level (*P* < 0.01), but water salinity only markedly influenced the expression levels of *nf-kb, il-1*β, and*tnf-*α (*P* < 0.05). The relative expression levels of *nf-kb, il-1*β, and *tgf*β*-1* had significant interactions between dietary cholesterol and water salinity (*P* < 0.05). Results of *t*-test analysis indicated that when fish fed with the cholesterol free diet (CHO), low salinity significantly up-regulated *nf-kb* and *tnf-*α expression levels. However, fish reared at low salinity and fed with CH1.6 diet (LCH1.6 group) had significantly lower expression levels of *il-1*β and *tnf-*α than in fish cultured at normal salinity (NCH1.6) (*P* < 0.05). Furthermore, one-way ANOVA showed that regardless of whether reared at normal or low salinity, fish fed dietary CH1.0 significantly down-regulated the expression levels of *nf-kb, il-1*β and *tnf-*α compared to dietary CH0.16 group (*P* < 0.05), while contrary results were found in anti-inflammatory cytokines, the expression levels of *il-10* and *tgf*β*-1* dramatically activated with the increase of dietary cholesterol level (*P* < 0.05), except for the expression level of*tgf*β*-1* in fish reared at normal water salinity (*P* > 0.05).

## Discussion

Adaptation of organism to the external environment is the basis of healthy existence (30). In aquatic animals, development and growth take place following characteristic steps for each species. Generally, it's affected by dietary nutrition, but it can also be under control of environmental factors directly, such as water salinity and temperature etc. (3). Besides, due to bony fishes having the ability to synthesize cholesterol (19), the need for exogenous cholesterol is still being discussed. In this study, the effects of dietary cholesterol level on growth performance and water salinity of *A. schlegelii* was evaluated. The results of two-way ANOVA showed that FBW, WG, and SGR of *A. schlegelii* were significantly affected by dietary cholesterol level and water salinity, but were not by interaction effects, suggesting that fish growth was influenced by dietary cholesterol and water salinity independently. Boeuf and Payan concluded that marine fish present higher developmental or growth rates at lower salinity, for juveniles of many fish species, opt for intermediary salinity conditions of the brackish water and grow optimally in estuaries and costal systems (3). This conclusion has also been confirmed in this study, the results of *t*-test analysis indicated that when fed the same dietary cholesterol level, fish reared at low salinity exhibited higher values of FBW, WG, and SGR than those in normal salinity. In agreement, Mozanzadehet al. reported that FBW, WG, and SGR were markedly enhanced when Asian seabass (*Lates calcarifer*) were reared at low salinity (6~12 psu), increasing water salinity over 12‰ gradually decreased growth parameters (31); a similar phenomenon was also found in sea bream (*Sparus sarbu*) (32) and red seabream (*Pagruspagrus*) (33). Growth performance improved by lower salinity compared to normal sea water was speculated that low water salinity could affect the stimulation of osmoregulatory hormones related to growth, but further studies are needed. Regarding the effect of dietary cholesterol level on growth performance, one-way ANOVA showed that regardless of whether reared at normal or low salinity, fish fed with 1% cholesterol (CH1.0) significantly increased growth performance compared to CH0.16 diet group, while no significant differences were found between CH0.16 and CH1.6 groups. Generally, bony fish have the capacity to synthesize cholesterol and, hence, it seems not necessary to supplement cholesterol in diet. However, a previous study indicated dietary soybean meal (SBM) at high level supplemented with cholesterol (0.6~1.2%) can promote the growth of rainbow trout (*Oncorhynchus mykiss*) (34). Similar results were found in studies on channel catfish (*Ictalurus punctatus*) (35), Japanese flounder (*Paralichthysolivaceus*) (36) and turbot (*Scophthalmus maximus*) (37). Besides, different results were found in Nile tilapia (*Oreochromis niloticus*) reared at brackish water, which indicated that dietary cholesterol supplementation does not substantially improve the growth of *O. niloticus*, and high dietary cholesterol level (over 0.8%) could lead to various negative effects (38). The findings of the present study suggest that the dietary 1% cholesterol supplementation could promote the growth performance of *A. schlegelii* at two levels of water salinity (23 psu and 5 psu); it might due to high level of soybean meal ingredients (soybean protein concentrate and SBW) were used in this study, and led to inadequate endogenous production of cholesterol.

The osmotic regulation function of gills is mainly mediated by gill mitochondrial chloride cell (MRC), which regulates proteins and hormones involved in osmotic pressure balance of the organism, such as NKA and cortisol, by absorbing and secreting Na^+^, K^+^, Cl^−^, etc., to maintain the osmotic pressure balance of organism (39, 40). The results of two-way ANOVA showed that most of serum ionic concentrations (Na^+^, Cl^−^, K^+^, and Mg^2+^) as well as hepatic NKA activity and related gene expression levels (*nkaa, ncc*and *ostf1*) of osmoregulation had significant interaction between dietary cholesterol level and water salinity. This is the first study that discovered the interaction between salinity and cholesterol on osmotic pressure regulation in euryhaline marine fish. When fed the same diet, fish cultured at low salinity significantly decreased the concentrations of Na^+^ (LCH0.16 and LCH1.6 groups) and Cl^−^ (LCH1.0 and LCH1.6 groups) in serum, while the contents of K^+^ (LCH0.16 and LCH1.6) and Mg^2+^ (LCH0.16) were increased, besides, no significant differences were found in serum Ca^2+^ content. These results are partially consistent with previous studies in pompano (*Trachinotus marginatus*) (41) and steelhead trout (*Oncorhynchus mykiss*) (11), suggesting that during euryhaline marine fish subjected to low salinity, due to hyper-osmoregulation and passive osmotic influx of water and, hence, resulted in diffusive loss of ions, which are mainly reflected in the variation of contents with serum Na^+^ or Cl^−^ (42). When fish were reared at low salinity, dietary CH1.0 markedly promoted serum Na^+^ concentration but decreased K^+^ and Mg^2+^content in serum when compared to CH0.16 diet. We speculate that dietary cholesterol in euryhaline marine fish may promote the absorption or retention of Na^+^ in serum to maintain the osmotic pressure balance. Cortisol is a corticosteroid produced by interrenal tissues of teleost species and involved in osmotic pressure regulation (43). In the present study, cortisol concentration in serum was determined, and results showed that when fish were fed the diet supplemented with cholesterol, and reared at low salinity, they had significantly higher cortisol content than at normal water salinity, but no significant differences were found between NCH0.16 and LCH0.16 treatments, showing that osmotic regulation was actively regulated by a combined effect of both cholesterol nutrition and low salinity. Furthermore, NKA is one of the important ion transporters, playing a vital role in osmotic pressure regulation and acting as a sodium pump that activates K^+^ inflow and Na^+^ outflow in sea water (43). Herein, NKA activity and its mRNA expression level in gills were analyzed, low salinity increased NKA activity occurred in LCH0.16 and LCH1.6 groups compared to those in fish reared at normal salinity and fed with the same diet, which showed positive correlation with cortisol. Moreover, previous studies have shown that Ncc plays a major role in osmotic pressure regulation in teleost species and expression of *ostf1* could be increased with extended exposure to a low-salinity (44). In the current study, hepatic *nka*α, *ncc*, and *ostf1* expression levels were varied with dietary cholesterol level and water salinity. In general, fish fed CH1.0 diet and cultured at low salinity showed higher expression levels than those in LCH0.16 and LCH1.6 groups; meanwhile, when fish fed with CH1.0 diet, low salinity markedly increased the gene expression levels of *nkaa, ncc*, and *ostf1* compared to fish reared at normal water salinity, which might be beneficial to maintain the osmotic pressure balance of body in low salinity. We speculate that those might be connected with the role of cholesterol, as a key constituent of cytomembrane, it can affect the osmoregulatory capacity of membrane bound proteins, which are participating in ion transport of gill cells (45, 46). Interestingly, Roy et al. previously reported that improved osmoregulatory capacity would result in less expenditure of energy directed toward regulation of hemolymph osmolality and, hence, could obtain better survival and growth (47). To some extent, this is consistent with the result of optimum growth performance obtained in this study in the fish that were fed the diet supplemented with 1% cholesterol.

Cholesterol is an integral component of cell membranes and it also serves as a precursor to important metabolites such as steroid hormones and bile acids (48). 3-hydroxy-3-methylglutaryl-CoA reductase (HMGCR) is considered as the most important rate-limiting enzyme to synthesize cholesterol fromacetyl-CoA. Moreover, cholesterol 7a-hydroxylase (CYP7A1) is the vital enzyme for primary bile acid synthesis and consequently promotes cholesterol catabolism (48). In this study, when fish reared at the same water salinity, one-way ANOVA showed that whether HMCRG activity or *hmcgr* expression level in liver were all decreased with the increase of dietary cholesterol level, but opposite results were found in TBA contents in the liver and intestine as well as hepatic *cyp7a1* expression level, which were reduced dramatically by dietary cholesterol supplementation. These results were consistent with previous studies in Atlantic salmon (*Salmo salar* L.), turbot (*Scophthalmusmaximus* L.), and rainbow trout (*Oncorhynchus mykiss*), where fish fed with high levels of cholesterol supplementation accelerate *cyp7a1* expression level and reduce HMGCR activity (34, 46, 48), showing that high levels of dietary cholesterol supplementation led to the suppression of cholesterol biosynthesis and increased conversion into bile acids. Interestingly, when fed the same diet, low salinity increased the HMCGR protein activity and gene expression level, as well as down regulation of *cyp7a1* when compared to normal salinity, which suggested that more cholesterol may need to be synthesized for energy to accommodate inlow salinity of marine fish. This speculation is also confirmed in the contents of cholesterol in serum and liver that high cholesterol level was maintained by low salinity. Both cholesterol and bile acid metabolism are controlled by nuclear receptors liver X receptor (LXR) and the farnesoid X receptor (FXR) and, generally, *cyp7a1* is positively regulated by LXR when serum cholesterol levels are high and negatively regulated by FXR when serum cholesterol levels are low (48, 49). Indeed, these were confirmed in this study that contrary regulatory expression pattern of *lxr* and *fxr* were observed, and *cyp7a1* expression level was down-regulated by *fxr*. The present data indicated that when fed the same diet, fish reared at low salinity markedly down -regulated *lxr* but up-regulated *fxr* expression level, which strongly supported the speculation that low salinity promotes cholesterol synthesis but inhibits cholesterol catabolism and, hence, the interactions between dietary cholesterol level and water salinity are significant. Furthermore, a previous study reported that *fxr* is activated by bile acids, and decreased intracellular bile acid levels by down-regulation of *cyp7a1* and up-regulation of *abcb11* in the liver (48). A consistent result was also found in the present study, but did not show any statistical differences among water salinity treatments, showing that water salinity has no impact on cholesterol transporters membrane of *abcb11* of *A. schlegelii*.

Fatty acids, especially n-3 LC-PUFAs are functionally important in responding to accommodating salinity change in aquatic animals. Tocher et al. concluded that fish fed HUFA-rich (highly unsaturated fatty acid) diets to a salinity stress could enhance osmoregulatory ability, particularly activate the activity of the NKA pump in gills (50). Moreover, a previous study has revealed that n-3LCPUFA can increase the unsaturation of cell membranes required to guarantee the regular operation of membrane proteins and ion transport (18). The present data showed that low salinity and dietary cholesterol supplementation increased the accumulation of n-3 PUFA, n-3 LC-PUFA, and DHA contents in liver, confirming that n-3 PUFA plays a vital role in adapting to a low salinity environment. Interestingly, we noticed that dietary cholesterol supplementation showed significantly higher fatty acid contents in liver, especially in fish cultured at low salinity. Unfortunately, there are limited studies that focused on the effects of dietary cholesterol on fatty acid synthesis at low salinity environment. In addition, the heat map clearly showed that the treatments of LCH1.0 and LCH1.6 clustered together, but LCH0.16 group was in a single cluster. Besides, PCA score plot and loading plot of fatty acids composition of liver revealed that n-3 PUFA, n-3 LC-PUFA, EPA, and DHA were at the upper right of PC1, which were highly correlated with the LCH1.0 diet group. Combined with the above results, our study suggested that moderate dietary cholesterol level (1% cholesterol in this study) could improve the accumulation of fatty acids in liver and, hence, these fatty acids might be transported to osmotic pressure to regulate the permeability of cell membrane and maintain ionic balance. Moreover, these results are consistent with a study previously reported by Fungwe et al. that an effect of dietary cholesterol to enhance the hepatic fatty acid synthesis rate (51). Additionally, fatty acid elongases and desaturases including elongase of *elovl5* and *fads2* are regarded as key enzymes in LC-PUFA biosynthesis in fish (52, 53). Hence, in order to further explore the role of dietary cholesterol in promoting fatty acid deposition, *elovl5* and *fads2* expression levels were measured. The present data showed that low salinity displayed significantly higher *elovl5* and *fads2* expression level than those in normal salinity. Besides, fish fed dietary CH1.0 showed higher expression levels of*elovl5* and *fads2* than other two dietary treatments at both water salinity. These results suggested that dietary 1% cholesterol supplementation could activate the expression levels of *elovl5* and *fads2* and, hence, promote LC-PUFA accumulation in liver. Furthermore, in mammals, it was previously reported that sterol regulatory element-binding protein 1c (SREBP-1c) mainly acts to activate the expression of genes involved in fatty acid synthesis, including fatty acid synthase, Fad, and Elovl (54, 55). Meanwhile, Srebp-1 and peroxisome proliferator-activated receptor α (Pparα) are involved in fatty acid biosynthesis *via* regulating *fads2* in fish, and the regulatory activity may be responsible for differential LC-PUFA biosynthesis abilities among fishes that have adapted to different ambient salinity (56). Interestingly, *srebp-1c* and *ppar*α showed the same pattern, where when fish reared at the same water salinity, dietary CH1.0 showed higher expression levels of these two genes than other treatments, consistent with the results of *elovl5* and *fads2* in this study, indicating that dietary CH1.0 promoted LC-PUFA accumulation might mediated by *srebp-1c* and *ppar*α regulation of *elovl5* and *fads*, and further studies are needed.

The adaptation of teleost fish to low salinity environment can also be reflected by the oxidative stress of liver (15); hence, herein, we explored the influence of dietary cholesterol level and water salinity on oxidative and antioxidant parameters in liver. In normal cell metabolism of aerobic animals, reactive oxygen species (ROS) will be produced naturally, leading to cell and tissue damage (57). Under normal physiological conditions, there is a balance between the generation and removal of ROS (58), but this balance could be destroyed by changes of environmental factors, for instance, salinity. The present study revealed that low salinity significantly increased MDA content and reduced the activities of GSH-px, SOD and T-AOC in the liver of fish, suggesting that low salinity stress could result in the oxidative stress. However, MDA content in fish reared under low salinity decreased with the increasing of dietary cholesterol levels. Besides, SOD and GSH-px activities as well as GSH content were significantly increased by dietary CH1.0 compared to LCH0.16 group, indicating that fed diet supplemented with moderate level of cholesterol (1% in the present study) could enhance antioxidant capacity in response to low salinity stress of fish. To some extent, these findings were in agreement with a previous study in rainbow trout (*Oncorhynchus mykiss*), where fish fed diets supplemented with 0.9 or 1.2% cholesterol showed significantly higher antioxidant capacity than the control group (0% cholesterol diet) (34). A previous study in tongue soles (*Cynoglossussemilaevis*) has demonstrated that low salinity could up-regulate the mRNA expression of immune-oxidative stress, and affecting the function of antioxidant enzyme system in liver (59). Indeed, the present study confirmed that low salinity displayed lower mRNA expression levels of antioxidant genes (*gpx, Cu-Zn sod*, and *Mn sod*) compared to normal salinity, except for *gpx* in dietary CH1.0 groups. A noticeable result is that antioxidant genes (*gpx* and *Mn sod*) were significantly up-regulated by fish fed with dietary CH1.0, which was further confirmed that dietary moderate level of cholesterol could activate antioxidant capacity at low salinity. Overall, the findings of this study indicated that fish cultured at low salinity and fed dietary CH1.0 could activate the antioxidant system in the liver, suggesting that 1% cholesterol supplementation could alleviate hepatic oxidative stress injury of *A. schlegelii*when reared at low salinity.

Numerous environmental (salinity, temperature, oxygen etc.) and physiological insults, as well as nutrient fluctuations, disrupt the endoplasmic reticulum protein-folding environment to cause protein misfolding and accumulation of misfolded proteins, referred to as ER stress (60). A previous study reported that with the increase of cellular oxidative stress, damaged proteins gradually accumulate in the endoplasmic reticulum cavity, triggering unfolded protein response (UPR) (61). The UPR signal network is mediated by three sensing proteins across the endoplasmic reticulum membrane, termed asinositol requiring enzyme- 1 α(Ire1α), protein kinase R–like endoplasmic reticulum kinase (Perk) and activating transcription factor 6 (Atf6) (62). Normally, Ire1α binds to and is inhibited by the chaperone protein glucose regulated protein 78 (Grp78) (60). When unfolded or misfolded proteins aggregate in the endoplasmic reticulum, Ire1α is activated and resulting related reactions (63). In this study, two-way ANOVA showed that significant interactions between dietary cholesterol level and water salinity were recorded in*ire1*α, *atf6, xbp1*, and *grp78* expression levels. As expected, when fish fed with cholesterol free diet and cultured at low salinity, *ire1*α*, atf6*, and *xbp1* expression levels were significantly up-regulated by low salinity, confirming that low salinity could cause ER stress in liver of fish. In turn, when fish reared at low salinity and fed dietary CH1.0 down-regulated markedly of *grp78, ire1*α*, atf6*, and *xbp1* expression levels compared to LCH0.16 treatment, which suggested dietary 1% cholesterol supplementation could alleviate ER stress caused by low salinity through suppressing the mRNA expression of ER stress related genes in liver.

A previous study has shown that salinity stress could limit the immune system function of fish, leading to the decline of immune function (64). Nuclear factor kappa B (Nf-κb) is a vital nuclear transcription factor, when NF-κB is activated, it transfers into the nucleus and promotes the expression of pro-inflammatory cytokines, including *tnf-*α and *il-1*β (56, 65). Besides, Tnf-α is processed as a membrane-bound protein from which the soluble active factor is cleaved by using the enzyme Tnf-α converting enzyme and *Il-1*β is one of the important pro-inflammatory cytokines produced by phagocytes (66). Moreover, in mammals, IL-10is an effective anti-inflammatory cytokine, regulating and inhibiting the expression of pro-inflammatory cytokines, contributing to resolve infection normally and reducing tissue damage caused by inflammation (67). Thus, the influence of dietary cholesterol level and water salinity on inflammatory response of liver were also analyzed in the present study. Results indicated that fed CH0.16 diet, low salinity up-regulated dramatically the expression levels of *tnf-*α and *nf-*κ*b*, indicating that inflammation response in liver could be caused in fish reared at low salinity, whereas no significant differences were found in anti-inflammatory cytokines. This was supported in Nile tilapia, which concluded that ambient salinity change resulted in inflammation of liver (68). Cholesterol is a component of lipids, which provide the fish with energy and essential fatty acids to relieve stress (69). Interestingly, regardless of whether reared at normal or low salinity, dietary CH1.0 displayed lower gene expression levels of *nf-*κ*b* and pro-inflammatory cytokine (*tnf-*α and *il-1*β*)* in lever than dietary CH0.16 group. Meanwhile, no significant differences of *tnf-*α, *il-1*β, and *nf-*κ*b* expression levels were observed between NCH0.16 and LCH0.16 groups, which indicated that dietary 1% cholesterol supplementation could alleviate inflammation caused by low salinity. Similar results were found in grass carp (*Ctenopharyngodonidella*), whereoptimal dietary cholesterol (~0.8% cholesterol) down-regulated the mRNA expression level of pro-inflammatory cytokines, whereas anti-inflammatory cytokines were down-regulated. Overall, the present results indicated that fish cultured at low salinity showed an activated inflammatory response in liver, this probably attribute to accelerate the release of cytokines such as *tnf-*α and *nf-*κ*b* in low salinity. Besides, our study suggested that moderate dietary cholesterol level (1% cholesterol) might alleviate inflammatory response caused by low salinity stress of *A. schlegelii*.

## Conclusion

**Figure 7:**
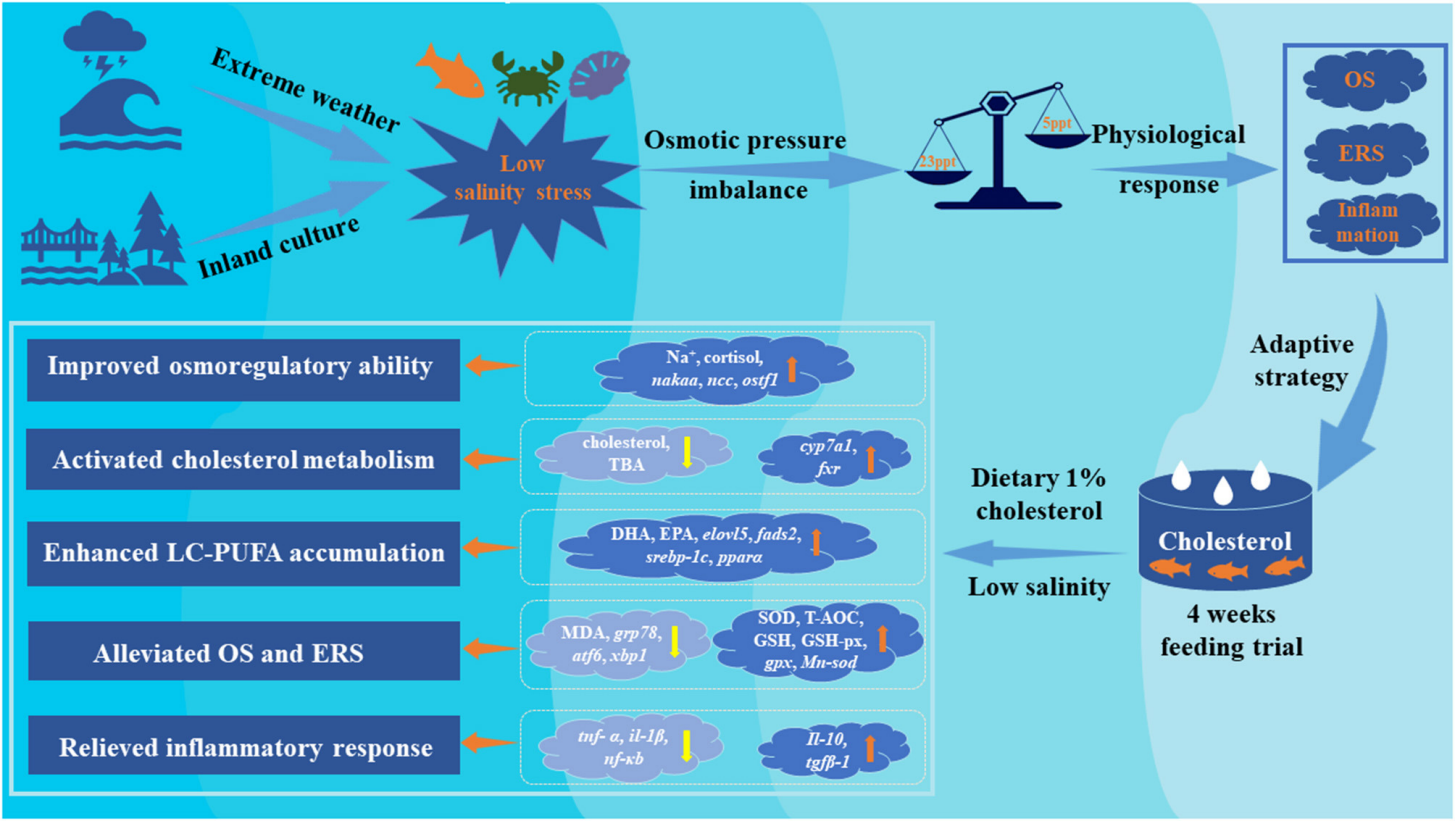
Proposed regulatory strategies of dietary cholesterol supplementation on adaptive of euryhaline marine fishes to low salinity environment. Yellow arrows represent promotion/up-regulation, orange arrows represent repression/down-regulation.

In conclusion, the present study suggested that dietary 1% cholesterol supplementation can promote growth performance of *A. schlegelii* in two water salinity (23 psu and 5 psu). Osmotic regulation was actively regulated by a combined effect of both cholesterol nutrition and low salinity, and fish fed the diet containing 1% cholesterol is beneficial to maintain osmotic pressure balance in response to low salinity environment. The current study revealed dietary cholesterol level and water salinity had significant interaction that low salinity promotes cholesterol synthesis but inhibits cholesterol catabolism of fish. Our study suggested that dietary 1% cholesterol supplementation can enhance the accumulation of fatty acids (especially n-3 LC-PUFA) in liver by activated key gene expression levels related to LC-PUFA biosynthetic pathway and, hence, regulate the permeability of cell membrane and maintain ionic balance. Low salinity caused various adverse effects including OS and ER stress in liver and, hence, triggering inflammatory response. Interestingly, our findings also indicated that dietary 1% cholesterol supplementation can alleviate these adverse effects by activating antioxidant parameters, suppressing expression levels of ER stress and pro-inflammatory makers. These findings provide further insight and understanding of the underlying adaptive strategy of euryhaline fishes to salinity stress, and confirmed it was dramatically affected by cholesterol nutrition (Figure 7).

## Data Availability Statement

The original contributions presented in the study are included in the article/Supplementary Materials, further inquiries can be directed to the corresponding author.

## Ethics Statement

The animal study was reviewed and approved by the Animal Research Institute Committee of Ningbo University, China.

## Author Contributions

QZ and MJ: conceptualization, methodology, validation, and supervision. YB, XL, and YS: formal analysis. LJ and ZW: resources. YB: writing—original draft. MJ: writing—reviewing and editing. All authors contributed to the article and approved the submitted version.

## Funding

This research was supported by National Key R&D Program of China (2018YFD0900400), National Natural Science Foundation of China (31802303), Fundamental Research Funds for the Provincial Universities of Zhejiang (SJLY2021007), Scientific Research Foundation of Ningbo University (XYL20007), Key Research Program of Zhejiang Province of China (2018C02037), and Zhejiang Aquaculture Nutrition & Feed Technology Service Team (ZJANFTST2017-2). This research was also sponsored by the K. C. Wong Magna Fund in Ningbo University.

## Conflict of Interest

The authors declare that the research was conducted in the absence of any commercial or financial relationships that could be construed as a potential conflict of interest.

## Publisher's Note

All claims expressed in this article are solely those of the authors and do not necessarily represent those of their affiliated organizations, or those of the publisher, the editors and the reviewers. Any product that may be evaluated in this article, or claim that may be made by its manufacturer, is not guaranteed or endorsed by the publisher.
