# Supplementary material for: A New Insight Into the Underlying Adaptive Strategies of Euryhaline Marine Fish to Low Salinity Environment: Through Cholesterol Nutrition to Regulate Physiological Responses
Source: Front Nutr. 2022 Apr 14;9:855369. doi: 10.3389/fnut.2022.855369 (PMC9097951; doi:10.3389/fnut.2022.855369)
Supplement: Supplementary file 1 [file Table_1.DOCX]

Supplementary Material

**Title**

**A new insight into the underlying adaptive strategies of** **euryhaline marine fishes to low salinity environment:** **Through** **the cholesterol nutrition** **to regulate physiological responses**

**Authors**

Yangguang Bao^1,2^, Yuedong Shen^1,2^, Xuejiao Li^1,2^, Zhaoxun Wu^1,2^, Lefei Jiao^1,2^, Jing Li^1,2^, Qicun Zhou^1,2^, Min Jin^1,2,^*

**Affiliations**

^1^Laboratory of Fish and Shellfish Nutrition, School of Marine Sciences, Ningbo University, Ningbo 315211, China

^2^Key Laboratory of Aquacultral Biotechnology Ministry of Education，Ningbo University,

Ningbo 315211, China

*** Corresponding author:**
Min Jin

[jinmin@nbu.edu.cn](mailto:jinmin@nbu.edu.cn)

**Contents:**

**Part 1. Supplementary Tables**

**Table** **S1.** Ingredients and proximate composition of the experimental diets (dry basis, g/ 100g).

**Table S2.** Fatty acid compositions (dry basis, %) of the experimental diets.

**Table S3.** Real-time quantitative PCR primers for cholesterol metabolism and osmotic regulation related genes of *Acanthopagrus schlegelii*.

**Table S4.** Fatty acid compositions (dry basis, %) of liver in *Acanthopagrus schlegelii* fed the experimental diets reared at different water salinity for 4 weeks.

**Part 2. References**

**Table S1**

Ingredients and proximate composition of the experimental diets (dry basis, g/ 100g).

| Ingredient (g/ 100g) | Dietary cholesterol levels | | |
| --- | --- | --- | --- |
|  | CH0.16 | CH1.0 | CH1.6 |
| Fish meal^a^ | 26.00 | 26.00 | 26.00 |
| Soybean protein concentrate^a^ | 10.00 | 10.00 | 10.00 |
| Soybean meal ^a^ | 16.00 | 16.00 | 16.00 |
| Wheat meal | 28.20 | 28.20 | 28.20 |
| Fish oil | 5.80 | 5.80 | 5.80 |
| Soybean oil | 5.80 | 5.80 | 5.80 |
| Cholesterol | 0.00 | 1.00 | 2.00 |
| Palmitic oil | 2.00 | 1.00 | 0.00 |
| Soybean Lecithin | 1.00 | 1.00 | 1.00 |
| Vitam mixture^b^ | 1.00 | 1.00 | 1.00 |
| Mineral mixture^c^ | 2.00 | 2.00 | 2.00 |
| Choline chloride | 0.20 | 0.20 | 0.20 |
| Ca(H_2_PO_4_)^2^ | 2.00 | 2.00 | 2.00 |
| Total | 100.00 | 100.00 | 100.00 |
| Nutrient levels (g/ 100g) ^d^ |  |  |  |
| Dry matter | 92.04 | 87.80 | 87.08 |
| Crude protein | 38.48 | 38.99 | 39.27 |
| Crude lipid | 19.26 | 18.91 | 19.86 |
| Crude ash | 9.73 | 9.66 | 9.68 |
| Energy | 21.65 | 21.63 | 22.07 |
| Cholesterol (actual value) | 0.16 | 1.00 | 1.60 |

^a^ These main ingredients were purchased from Ningbo Tech‐Bank Feed Co. Ltd (Ningbo, China). Fishmeal, crude protein, 710/ g kg, crude lipid, 108/ g kg; soybean meal, crude protein, 527 g/ kg, crude lipid, 19 g/ kg.

^b^ Vitamin mixture (mg/ 100g diet): D-Ca pantothenate, 120; inositol, 200; menadione, 60; nicotinic

acid, 100; pyridoxine hydrochloride, 60; riboflavin, 50; thiamin nitrate, 60; all-rac-a-tocopherol, 100; cyanocobalamin, 0.1; biotin, 6.0; folic acid, 10; retinyl acetate, 5000 IU; cholecalciferol, 2000 IU.

^c^ Mineral mixture (mg/ 100g diet): NaCl (99.5%), 767 mg; KCl (99.5%), 1916 mg; CuSO4·5H_2_O (99%) 117.188 mg; MgSO_4_·7H_2_O (99%), 6145 mg; FeSO4·7H2O 198.61 mg; CoSO_4_·7H_2_O 1667 mg; MnSO_4_·H_2_O 30.731 mg; Ca (IO_3_)_2_ (0.99%) 0.077 mg; Na_2_SeSO_3_ 0.779 mg.

^d^ Nutrient level was measured value (dry matter basis). All values are as g/ kg of diets

**Table S2**

Fatty acid compositions (dry basis, %) of the experimental diets.

| Items | Dietary cholesterol levels | | |
| --- | --- | --- | --- |
|  | CH0.16 | CH1.0 | CH1.6 |
| 14:0 | 2.43 | 2.33 | 2.32 |
| 16:0 | 23.76 | 16.15 | 12.55 |
| 18:0 | 5.40 | 5.79 | 5.77 |
| 20:0 | 0.44 | 0.51 | 0.52 |
| ∑SFA^1^ | 32.03 | 24.78 | 21.17 |
| 16:1n | 2.85 | 2.82 | 2.83 |
| 18:1n-9 | 12.24 | 11.48 | 11.57 |
| 20:1n-9 | 0.88 | 0.98 | 0.98 |
| 22:1n-11 | 0.19 | 0.22 | 0.24 |
| ∑MUFA^2^ | 16.16 | 15.50 | 15.62 |
| 18:2n-6 | 47.76 | 43.40 | 43.94 |
| 18:3n-6 | 0.08 | 0.10 | 0.10 |
| 20:2n-6 | 0.13 | 0.14 | 0.15 |
| 20:4n-6 | 0.56 | 0.64 | 0.65 |
| n-6PUFA^3^ | 48.53 | 44.27 | 44.83 |
| 18:3n-3 | 3.45 | 3.62 | 3.71 |
| 18:4n-3 | 0.93 | 0.93 | 0.98 |
| 20:4n-3 | 0.45 | 0.46 | 0.49 |
| 20:5n-3 | 3.38 | 3.64 | 3.77 |
| 22:5n-3 | 0.75 | 0.86 | 0.89 |
| 22:6n-3 | 6.94 | 7.28 | 7.57 |
| DHA/EPA | 2.05 | 2.00 | 2.01 |
| n-3PUFA^4^ | 15.91 | 16.79 | 17.41 |
| n-3LCPUFA^5^ | 11.52 | 12.23 | 12.72 |

^1^ SFA, saturated fatty acids; ^2^ MUFA, monounsaturated fatty acids; ^3^ n-6 PUFA, n-6 polyunsaturated fatty acids; ^4^ n-3 PUFA, n-3 polyunsaturated fatty acids; ^5^ n-3LCPUFA, n-3 long chain polyunsaturated fatty acids. Values are presented as the means ± SEM of three replicates (n= 3).

**Table S3**

Real-time quantitative PCR primers for cholesterol metabolism and osmotic regulation related genes of *Acanthopagrus schlegelii.*

| Gene | Nucleotide sequence (from 5′ to 3′) | Size (bp) | Accession no.  or Publication | Functions |
| --- | --- | --- | --- | --- |
| *hmgcr*^1^ | F: ACCCACCTTCACTCTGGATG  R: CTGACTCTCACGGGACAACT | 187 | OL689005 | Cholesterol metabolism |
| *cyp7a1*^2^ | F: ACACCACAGAGAACCTCCAC  R: CATCCACCTCCCAGTCGTCCTT | 168 | Tong et al. (1) | Cholesterol metabolism |
| *lxr^3^* | F: AACAGCCAAGAACGGACG  R: TTGAGGATGCCAAAACCC | 156 | OL689006 | Cholesterol metabolism |
| *fxr*^4^ | F: AGTTGGAGTAAGGAGTCGCC  R: GGTTGCGTTTGTTGGTTGTG | 169 | OL689007 | Cholesterol metabolism |
| *abcg*^5^ | F: CGAGGGTGGTCCTATTGG  R: GCGGTTGGTGGATGGTTACT | 126 | OL689008 | Cholesterol metabolism |
| *abcb11*^6^ | F: CTGGTGGGGTAGGTGAACTT  R: TCCTCCTACACACCGGACTA | 167 | OL689009 | Cholesterol metabolism |
| *ncc*^7^ | F: TTGCTGTTGTCGGCTCG  R: CCACGCTGGGTTTGTCC | 235 | OL321595 | Osmoregulation |
| *ostf1*^8^ | F: TTGTGGCCATCGACAACAAG  R: GATTGTCCAAGAGCACGTCC | 243 | OL321591 | Osmoregulation |
| *nkaα*^9^ | F: GTCGTCATCATCACCGGTTG  R: GATTCTCAGATCAGCGGGGA | 201 | EF621407 | Osmoregulation |
| *gpx^10^* | F: TCTGAAGTACGTCCGTCCTG  R: TCTCAAAGTTCCAGGCCACA | 247 | OL321587 | Antioxidation |
| *Mn-sod^11^* | F: TCTCTTTCTCGTAGCCCAGC  R: GCAAAGGGAGATGTGACAGC | 247 | OL321589 | Atioxidation |
| *Cu-Zn sod^12^* | F: CACGGTAAGAATCATGGCGG  R: TCTCCTCGTTGCCTCCTTTT | 202 | OL321588 | Antioxidation |
| *foxO1^13^* | F: GAAGCTGTTCCACGCATCAA  R: TTCACCAAGAGCAGAGGGAG | 150 | OL752705 | Antioxidation |
| *tnfα^14^* | F: GTCCTGCTGTTTGCTTGG  R: AATGGATGGCTGCCTTGG | 154 | AY335443 | Pro-inflammatory cytokine |
| *il-1β^15^* | F: CATCTGGAGGCGGTGAA  R: CGGTTTTGGTGGGAGGA | 231 | JQ973887 | Pro-inflammatory cytokine |
| *tgfβ-1^16^* | F: GGGTTTCCAACTTCGGC  R: TTGTGTCCGTGGAGCGT | 209 | Xue et al. (2) | Anti-inflammatory cytokine |
| *il-10^17^* | F: GTTCTGGGCAGCTGTAGAGG  R: CCCAGATAGAAGCCCAGGAT | 105 | MK922542 | Anti-inflammatiory cytokine |
| *nfκb^18^* | F: AGCCCAAGGCACTCTAGACA  R: GTTCTGGGCAGCTGTAGAGG | 154 | MK922543 | Nuclear transcription factor |
| *grp78^19^* | F: AACCAGCTGACCTCTAACCC  R: ATGTCTTCATCTGGCCACCA | 164 | MT451934 | ERS pathway |
| *ire1α^20^* | F: AGAGGTCTTGGGTCATGGTG  R: AGAGGTCTTGGGTCATGGTG | 181 | OL361769 | ERS pathway |
| *atf6^21^* | F: CCTGTTGGGTTTCTCCTCAG  R: CCGTTACTTCACAGTCAATCTGC | 222 | MT512507 | ERS pathway |
| *xbp1^22^* | F: CCTGTTGGGTTTCTCCTCAG  R: TTCCTGTCTCTGGCTGTCTG | 235 | MW589390 | ERS pathway |
| *elovl4b^23^* | F: ATCCAGTTCCACGTGACCAT  R: TCCCATTTTCCTCCACCTCC | 222 | KU372150 | LC-PUFA biosynthetic pathway |
| *elvol5^24^* | F: TCGTACTTCGGTGCCTCCCT  R: GGCCATATGACTGCAAATATTGC | 176 | KU372149 | LC-PUFA biosynthetic pathway |
| *fads2^25^* | F: AGCCAGGACCGAAATAAAA  R: AGGTGGAGGCAGAAGAACA | 113 | KX058437 | LC-PUFA biosynthetic pathway |
| *β-anctin* | F: ACCCAGATCATGTTCGAGACC  R: ATGAGGTAGTCTGTGAGGTCG | 212 | Jiao et al. (3) | Housekeeping gene |

^1^ *hmgcr*, 3-hydroxy-3-methyl-glutaryl-CoA reductase.

^2^ *cyp7a1*, cytochrome P450 family 7 subfamily A member 1.

^3^ *lxr*, liver X receptor.

^4^ *fxr*, farnesoid X receptor.

^5^ *abcg5*, ATP binding cassette G5.

^6^ *abcb11*, ATP binding cassette B11.

^7^ *ncc*, Na^+^/Cl^−^ cotransporter.

^8^ *ostf1*, osmotic stress transcription factor 1.

^9^ *nkaα*, sodium/potassium ATPase alpha.

^10^ *gpx,* glutathione peroxidase.

^11^ *Mn-sod,* manganese superoxide dismutase.

^12^ *Cu-Zn sod,* copper/zinc Superoxide dismutase.

^13^ *foxO1,* forkhead O1.

^14^ *tnf-a,* tumor necrosis factor α.

^15^ *il-1β,* interleukin-1β.

^16^ *tgfβ-1,* transforming growth factor β.

^17^ *il-10,* interleukin-10.

^18^ *nf-κb,* nuclear factor kappa b.

^19^ *grp78,* glucose regulated protein 78.

^20^ *ire1α,* inositol requiring enzyme-1.

^21^ *atf6,* activating transcription factor 6.

^22^ *xbp1,* X-box binding protein 1.

^23^ *elovl4b,* elongase of very long chain fatty acid 4b.

^24^ *elvol5,* elongase of very long chain fatty acid 5.

^25^ *fads2,* fatty acid desaturase.

**Table S4**

Fatty acid compositions (dry basis, %) of liver in *Acanthopagrus schlegelii* fed the experimental diets reared at different water salinity for 4 weeks.

| Items | Dietary treatments in normal water salinity | | |  | | Dietary treatments in low water salinity | | |
| --- | --- | --- | --- | --- | --- | --- | --- | --- |
|  | NCH0.16 | NCH1.0 | NCH1.6 |  | LCH0.16 | | LCH1.0 | LCH1.6 |
| 14:0 | 7.48±0.27^A^ | 9.26±0.09^B^ | 8.64±0.31^B^ | | 6.86±0.08^a^ | | 9.86±0.16^c #^ | 8.78±0.00^b^ |
| 16:0 | 49.76±1.37^B^ | 62.31±0.62^C^ | 45.94±0.48^A^ | | 47.44±3.03^a^ | | 56.33±0.91^b ##^ | 48.11±0.25^a #^ |
| 18:0 | 25.99±0.32^B^ | 29.11±1.12^C^ | 20.85±0.05^A^ | | 20.52±0.69^a ##^ | | 25.22±0.55^b #^ | 23.45±0.01^b ##^ |
| 20:0 | 0.85±0.02 | 0.92±0.01 | 0.83±0.07 | | 0.73±0.13 | | 0.81±0.03^#^ | 0.84±0.01 |
| ∑SFA^1^ | 85.86±0.66^B^ | 102.65±0.03^C^ | 76.26±0.77^A^ | | 75.55±3.87^a^ | | 92.22±1.60^b ##^ | 81.19±0.23^a ##^ |
| 16:1n | 8.14±0.20^A^ | 10.69±0.05^C^ | 9.35±0.28^B^ | | 7.72±0.15^a^ | | 10.67±0.82^b^ | 10.02±0.03^b^ |
| 18:1n-9 | 79.71±0.07^A^ | 98.81±0.51^B^ | 81.21±0.70^A^ | | 74.65±1.73^a #^ | | 96.66±2.36^c^ | 88.83±0.09^b ##^ |
| 20:1n-9 | 2.45±0.10 | 2.68±0.09 | 2.62±0.18 | | 1.34±0.03^a ##^ | | 2.97±0.01^c^ | 2.51±0.00^b^ |
| 22:1n-11 | 0.72±0.03 | 0.79±0.05 | 0.78±0.06 | | 0.74±0.04 | | 0.78±0.01 | 0.86±0.03 |
| ∑MUFA^2^ | 91.01±0.34^A^ | 112.98±0.43^C^ | 94.93±0.05^B^ | | 84.45±1.62^a #^ | | 111.09±2.83^c^ | 102.22±0.09^b ##^ |
| 18:2n-6 | 49.01±0.04^A^ | 55.51±0.30^B^ | 54.55±1.85^B^ | | 41.41±0.11^a ##^ | | 60.97±1.19^c #^ | 57.10±0.41^b^ |
| 18:3n-6 | 6.44±0.24^B^ | 5.71±0.23^B^ | 3.98±0.10^A^ | | 8.19±0.52^#^ | | 6.74±0.28^#^ | 7.45±0.03 ^##^ |
| 20:2n-6 | 2.27±0.09 | 2.52±0.16 | 2.57±0.21 | | 1.91±0.20^a^ | | 2.61±0.08^b^ | 2.30±0.03^ab^ |
| 20:4n-6 | 2.87±0.10 | 3.34±0.02 | 2.94±0.19 | | 3.67±0.18^b #^ | | 2.80±0.06^a ##^ | 2.90±0.10^a^ |
| n-6PUFA^3^ | 60.58±0.09^A^ | 67.6±0.12^C^ | 66.01±0.39^B^ | | 55.18±0.77^a ##^ | | 73.12±1.32^b #^ | 69.75±0.35^b ##^ |
| 18:3n-3 | 5.61±0.24^A^ | 6.66±0.07^B^ | 6.65±0.11^B^ | | 4.80±0.05^a #^ | | 7.72±0.27^c #^ | 6.80±0.07^b^ |
| 18:4n-3 | 1.64±0.03^B^ | 1.74±0.05^B^ | 1.47±0.03^A^ | | 1.48±0.00^a ##^ | | 1.90±0.03^b^ | 1.97±0.05^b ##^ |
| 20:4n-3 | 2.7±0.09 | 2.65±0.14 | 2.45±0.15 | | 2.14±0.03^##^ | | 2.68±0.15 | 2.58±0.02 |
| 20:5n-3 | 7.08±0.25 | 7.92±0.27 | 7.36±0.33 | | 6.61±0.06^a^ | | 8.45±0.02^c^ | 7.81±0.08^b^ |
| 22:5n-3 | 5.32±0.07 | 4.89±0.18 | 4.62±0.24 | | 4.41±0.01^a ##^ | | 4.81±0.05^b^ | 4.51±0.06^a^ |
| 22:6n-3 | 28.91±0.30^B^ | 31.53±0.14^C^ | 27.74±0.23^A^ | | 30.27±0.57^b^ | | 27.22±0.62^a ##^ | 29.86±0.39^b #^ |
| n-3PUFA^4^ | 51.27±0.48^A^ | 55.82±0.55^B^ | 50.30±0.56^A^ | | 49.71±0.49^a^ | | 52.78±0.78^b #^ | 53.54±0.35^b ##^ |
| n-3LCPUFA^5^ | 44.53±0.04^B^ | 47.38±0.50^C^ | 41.74±0.12^A ##^ | | 43.43±0.54 | | 43.16±0.69 ^##^ | 44.76±0.34 ^##^ |
| Items | *P* values (two-way ANOVA) | | | | | | | |
|  | Cholesterol | | Salinity | | | | Cholesterol*salinity | |
| 14:0 | <0.01 | | 0.80 | | | | 0.02 | |
| 16:0 | <0.01 | | 0.11 | | | | 0.05 | |
| 18:0 | <0.01 | | <0.01 | | | | <0.01 | |
| 20:0 | 0.49 | | 0.18 | | | | 0.56 | |
| ∑SFA^1^ | <0.01 | | <0.01 | | | | <0.01 | |
| 16:1n | <0.01 | | 0.80 | | | | 0.37 | |
| 18:1n-9 | <0.01 | | 0.90 | | | | <0.01 | |
| 20:1n-9 | <0.01 | | <0.01 | | | | <0.01 | |
| 22:1n-11 | 0.09 | | 0.35 | | | | 0.50 | |
| ∑MUFA^2^ | <0.01 | | 0.73 | | | | <0.01 | |
| 18:2n-6 | <0.01 | | 0.86 | | | | <0.01 | |
| 18:3n-6 | <0.01 | | <0.01 | | | | <0.01 | |
| 20:2n-6 | 0.02 | | 0.15 | | | | 0.29 | |
| 20:4n-6 | 0.05 | | 0.48 | | | | <0.01 | |
| n-6 PUFA^3^ | <0.01 | | 0.04 | | | | <0.01 | |
| 18:3n-3 | <0.01 | | 0.32 | | | | <0.01 | |
| 18:4n-3 | <0.01 | | <0.01 | | | | <0.01 | |
| 20:4n-3 | 0.13 | | 0.17 | | | | 0.02 | |
| 20:5n-3 | <0.01 | | 0.33 | | | | 0.06 | |
| 22:5n-3 | 0.07 | | <0.01 | | | | 0.01 | |
| 22:6n-3 | 0.18 | | 0.42 | | | | <0.01 | |
| n-3 PUFA^4^ | <0.01 | | 0.33 | | | | <0.01 | |
| n-3 LCPUFA^5^ | <0.01 | | 0.06 | | | | <0.01 | |

^1^ SFA, saturated fatty acids; ^2^ MUFA, monounsaturated fatty acids; ^3^ n-6 PUFA, n-6 polyunsaturated fatty acids; ^4^ n-3 PUFA, n-3 polyunsaturated fatty acids; ^5^ n-3LCPUFA, n-3 long chain polyunsaturated fatty acids. Values are presented as the means ± SEM of three replicates (n= 3). *P* values of influences of salinity, dietary cholesterol level and their interaction on the relevant parameters are presented. “ABC” representing significant (*P*< 0.05) difference between dietary cholesterol levels in normal water salinity (23 psu) and “abc” representing significant (*P*< 0.05) difference between dietary cholesterol levels in low water salinity (5 psu) by performing one-way ANOVA. “#” representing significant (*P*< 0.05) difference between water salinity with same dietary cholesterol level and “##” representing highly significant (*P*< 0.01) difference between water salinity with same dietary cholesterol level by performing *t* test.

**References**

1. Tong, S., Wang, L., Kalhoro, H., Volatiana, J.A., Shao, Q. (2020). Effects of supplementing taurine in all‐plant protein diets on growth performance, serum parameters, and cholesterol 7α‐hydroxylase gene expression in black sea bream, *Acanthopagrus schlegelii*. Journal of the World Aquaculture Society. 51(4): 990-1001. doi: 10.1111/JWAS.12611.
2. Xue, L., Yang, Q., Xue, L., Yang, Q., Xiao, Z., Xue, L., Yang, Q., Xiao, Z., Li, L., Xue, L., Yang, Q.S., Xiao, Z., Li, L. (2008). Molecular characterization of myostatin in black seabream, Acanthopagrus schlegelii, DNA Sequence. 19: 217-223. doi: 10.1080/10425170701517564.
3. Jiao, B., Huang, X., Chan, C.B., Zhang, L., Wang, D., Cheng, C.H.K. (2006). The co-existence of two growth hormone receptors in teleost fish and their differential signal transduction, tissue distribution and hormonal regulation of expression in seabream. Journal of Molecular Endocrinology. 36, 23-40. doi: 10.1677/JME.1.01945.
